# HIC1 suppresses Tumor Progression and Enhances CD8^+^ T Cells Infiltration Through Promoting GSDMD‐induced Pyroptosis in Gastric Cancer

**DOI:** 10.1002/advs.202412083

**Published:** 2025-04-25

**Authors:** Mengjie Kang, Wenqi Du, Lina Ding, Mengdi Wu, Dongsheng Pei

**Affiliations:** ^1^ Department of Pathology School of Basic Medical Sciences Xuzhou Medical University 209 Tongshan Road Xuzhou Jiangsu 221004 China; ^2^ Department of Anatomy School of Basic Medical Sciences Xuzhou Medical University 209 Tongshan Road Xuzhou Jiangsu 221004 China

**Keywords:** cell pyroptosis, gastric cancer, GSDMD, HIC1, immune infiltration

## Abstract

Recently, immune checkpoint blockade treatment has made remarkable strides in combatting malignancies, including gastric cancer (GC). Nonetheless, the efficacy of immunotherapy in GC patients remains constrained, warranting further exploration of the underlying molecular mechanisms to improve therapeutic outcomes. Hypermethylated in cancer 1 (HIC1) is acknowledged as a transcriptional regulator crucial for multiple aspects of cell development, yet its role in antitumor immune responses remains incompletely understood. This investigation reveals a significant downregulation of HIC1 in gastric cancer, correlating with a less favorable prognosis. Overexpression of HIC1 promotes the initiation of cell pyroptosis. Mechanistically, gasdermin D (GSDMD), a pivotal executor of pyroptosis, is identified as a downstream target of HIC1 and activated by HIC1 at the transcriptional level. Subsequent cleavage of the GSDMD N‐terminal region punctures the cell membrane, instigating pyroptosis and releasing inflammatory factors. Furthermore, HIC1 augments the infiltration of CD8^+^ T cells to counteract immune evasion. The combinatorial approach of HIC1 overexpression with PD‐L1 antibody demonstrates a synergistic therapeutic impact in treating GC. Additionally, c‐Jun activation domain‐binding protein 1 (Jab1) mediates the ubiquitylation and proteasomal degradation of HIC1 at Lys^517^. Ultimately, these findings underscore the potential of HIC1 as a promising immunotherapeutic target for the treatment of GC.

## Graphical abstract

These findings elucidate the innovative role of HIC1 as a transcriptional activator in GC, driving the initiation of pyroptosis and enhancing CD8^+^ T cell infiltration, which has certain novelty and creative significance. Collectively, targeting HIC1 can present an appealing immunotherapeutic strategy to improve outcomes in GC patients.

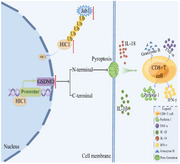

## 1 Introduction

Globally, gastric cancer (GC) ranks as the fifth most commonly diagnosed malignancy and the fourth predominant cause of cancer death, accounting for ≈768 000 deaths among 1 million cases in 2020.^[^ ^1^ ^]^ Interestingly, the prevalence of GC in young individuals has increased noticeably. And the great majority of patients were gradually diagnosed with GC at an advanced stage, suffering an inferior outcome.^[^ ^2^ ^]^ Recent advancements in medical immunology and molecular biology have sparked considerable interest in the field of immunotherapy. For instance, programmed death ligand‐1 (PD‐L1) binds to programmed death‐1 (PD‐1) on T cells, triggering T cell deactivation and enabling tumor cells to evade the host's immune response.^[^ ^3^ ^]^ GC‐derived exosomes boosted the accumulation of PD1+ TAM (tumor‐associated macrophage), which further compromised CD8^+^ T cells' effectiveness and obstructed GC advancement.^[^ ^4^ ^]^ Additionally, accumulating evidence indicates that the tumor microenvironment (TME) is not only pivotal in tumorigenesis but also intimately associated with the efficacy of immunotherapy.^[^ ^5^ ^]^ Derks et al. pointed out the heterogeneous nature of the tumor immune microenvironment in GC, emphasizing the important role of TME in the immunotherapeutic response to GC.^[^ ^6^ ^]^ Although immunotherapy has documented benefits for numerous carcinomas, it has not been equally conducive for all GC patients.^[^ ^7^ ^]^ Therefore, deciphering the underlying mechanisms of GC development and how GC cells fortify the antitumor immune response against malignancies could provide insights into pioneering biomarkers and comprehensive treatment strategies for GC.

Available evidence suggests that programmed cell death (PCD), including apoptosis, ferroptosis, and pyroptosis, exerts indispensable functions in regulating tumorigenesis, holding promise for cancer immunotherapies.^[^ ^7^ ^]^ Pyroptosis, which initially emerged in the 1980s, is characterized by cell expansion, membrane rupture, pore formation, as well as the generation of inflammatory cytokines such as interleukin‐1β (IL‐1β) and interleukin‐18 (IL‐18), leading to provoke robust immune responses.^[^ ^8^ ^]^ Additionally, studies claimed that gasdermin D (GSDMD) served as the executor of pyroptosis.^[^ ^9^ ^]^ There are two distinct inflammatory pathways involved in pyroptosis: canonical and noncanonical pathways. The activation of the canonical pathway primarily involves Caspase‐1, whereas the noncanonical pathway is dependent on Caspase‐4/5 in humans or Caspase‐11 in mice.^[^ ^10^ ^]^ In recent decades, a growing body of evidence has unraveled the intricate interplay between nonapoptotic programmed cell death and antitumor immunity. Notably, CD8^+^ T lymphocytes have been shown to induce a significant reduction in SLC7A11 expression, a protein responsible for cysteine uptake, glutathione synthesis, and ferroptosis induction.^[^ ^11^ ^]^ Moreover, in myeloid cells, inflammasome‐mediated pyroptosis demonstrated a profound link with both cancer progression and immunity.^[^ ^12^ ^]^ Wang et al. described that tumor cell pyroptosis remarkably augmented the infiltration percentage of CD8^+^ T and CD4^+^ T cells and the tumor regression was abolished in circumstances where T cells were depleted or in mice with immunological deficiencies.^[^ ^13^ ^]^ Hence, clarifying the intricate crosstalk between pyroptosis and immunity holds significant value for subsequent research endeavors.

HIC1, commonly referred to as hypermethylated in the Cancer 1 gene, occurred on chromosome 17p 13.3 and participated in an extensive spectrum of broad biological processes involving cell proliferation, cell metastasis, and motility.^[^ ^14^ ^]^ Moreover, it has been extensively acknowledged that HIC1 encoded a tumor suppressor gene that was generally silenced epigenetically in a variety of carcinomas, such as pancreatic cancer, breast cancer, prostate cancer, renal cell carcinoma, and laryngeal carcinoma.^[^ ^15^ ^]^ Additionally, HIC1 is also a member of BTB/POZ domain and C2H2Krüppel‐like Zinc fingers family, endowing it with the capacity to function as a transcriptional regulator function by binding to a certain motif.^[^ ^16^ ^]^ Recent literature has suggested that hypermethylation of the HIC1 promoter could serve as a pivotal factor driving its downregulation in gastric cancer.^[^ ^17^ ^]^ Beyond that, HIC1 may impede the progression of gastric cancer by preserving normal cellular metabolism and attenuating the activation of the mTOR signaling pathway.^[^ ^17b^ ^]^ Loss of HIC1 expression was profoundly linked to enhanced cell proliferation, diminished pyroptosis, and compromised antitumor immune responses, all of which collectively contributed to tumor progression and are predictive of an unfavorable prognosis in our study. Equally important, investigations have revealed the presence of additional post‐translational modification mechanisms of HIC1, such as SUMOylation, glycosylation, and acetylation.^[^ ^18^ ^]^ In this article, we highlighted the immediate interaction between HIC1 and c‐Jun activation domain‐binding protein‐1 (Jab1). Jab1, also called the fifth subunit of constitutive photomorphogenic‐9 (COP9) signalosome complex (CSN) which implicated in the regulation of protein stability, transcription, protein phosphorylation, and intracellular distribution.^[^ ^19^ ^]^ Although the role of HIC1 in various malignancies has been well‐documented, its precise mechanisms in gastric cancer remain inadequately elucidated. Consequently, it is of essential scientific significance to explore the correlation between HIC1 expression and gastric cancer pathogenesis.

In the current study, we demonstrated that HIC1 was downregulated and significantly correlated with a grim prognosis in GC. Functionally, HIC1 exerted an inhibitory effect on cell proliferation while promoting pyroptosis, characterized by membrane rupture and bubble formation. Subsequently, our findings discovered that HIC1 facilitated cell pyroptosis by transcriptionally activating the downstream target GSDMD, leading to an increase in tumor‐infiltrating CD8^+^ T cells. Eventually, we elucidated that endogenous Jab1 interacted with HIC1 proteins, and through mass spectrometry analysis, ubiquitination sites were identified. These findings underscored the potential of targeting HIC1 as a strategy to enhance therapeutic efficacy.

## 2 Results

### 2.1 HIC1 is Downregulated and Associated with Prognosis in GC

**Figure 1:**
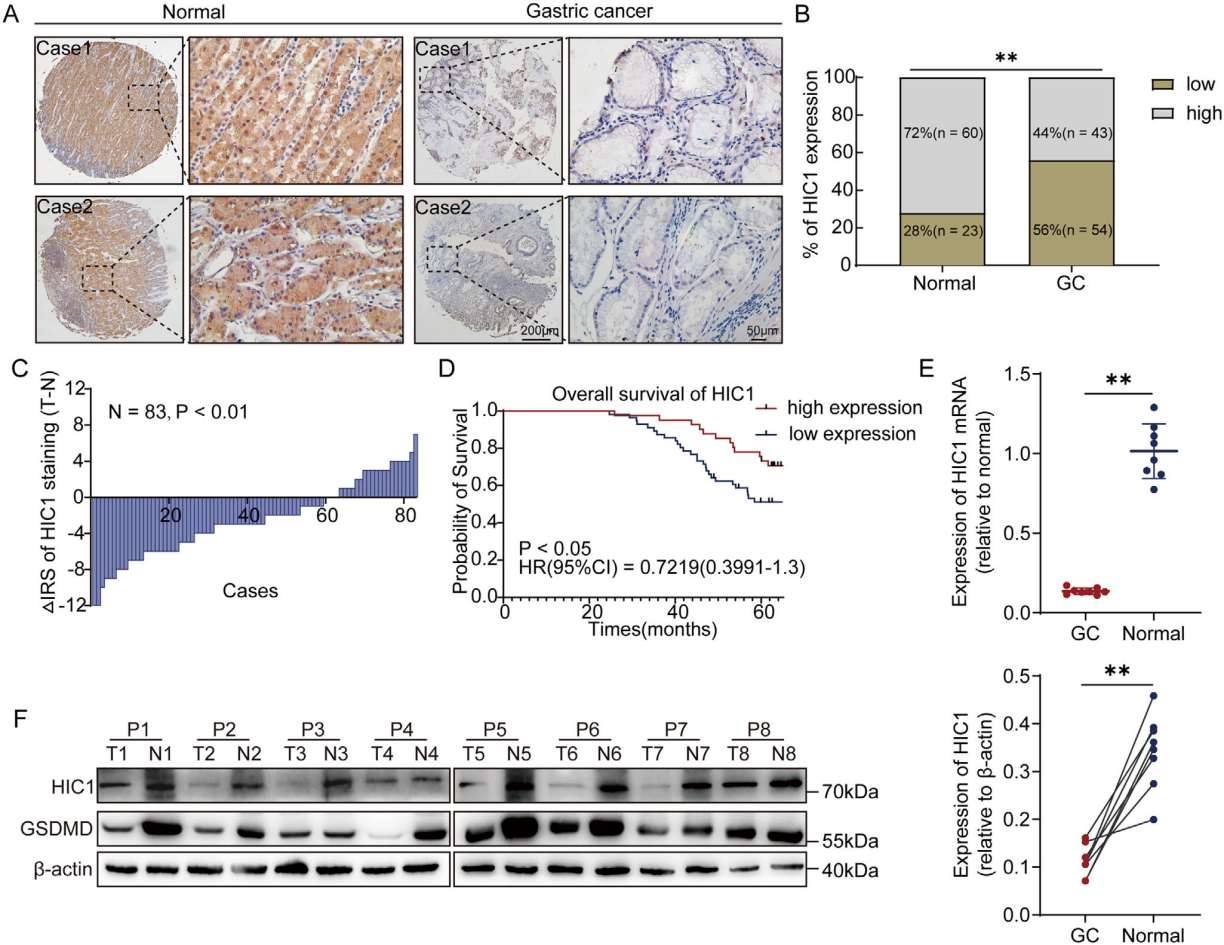
HIC1 is considerably downregulated in human GC tissues and predicts an inferior prognosis. (A) Representative microscopic images depicting HIC1 immunohistochemical staining in a gastric cancer tissue microarray (TMA) containing 83 GC tissues and 97 normal specimens were presented. (B) IHC statistical analysis revealed the percentage of HIC1 levels in GC tissues and normal tissues. (Normal group = 83, GC group = 97, **p < 0.01). (C) Based on IHC staining scores, the expression level of HIC1 were categorized into low and high groups. The disparity in the immunoreactivity score (IRS) (∆IRS = IRS_T_−IRS_N_) of HIC1 was demonstrated. (D) Overall survival curve according to the expression of HIC1. The significance of the *P* value was defined through the log‐rank test (*p <* 0.05, HR = 0.7219). (E,F) The protein and mRNA expression levels of HIC1 in eight patients with GC were accessed by western blot and RT‐qPCR assays. (“T” signified tumor and “N” signified normal). Data given are mean ± SD. **p < 0.01.

To elucidate the essential roles of HIC1 in the development of GC, we first identified the HIC1 expression levels by immunohistochemistry (IHC) staining in the GC tissue microarray encompassing 83 normal gastric mucosa and 97 GC tissues and found that the gene expression of HIC1 was statistically lower in GC tissues compared with normal gastric mucosa (**Figure** **1**A–C). Based on the staining results, GC patients were divided into high‐expression and low‐expression groups. The patients with low expression of HIC1 demonstrated a considerably reduced probability of overall survival (Figure 1D). An analysis of the correlation between HIC1 expression and various clinicopathological parameters in GC patients was presented in Table S1, Supporting Information. Besides, to investigate whether HIC1 expression could serve as an independent prognostic factor for GC, we performed a univariate and multivariate Cox regression analysis adjusting for other clinical variables such as age, gender, and tumor size (Table S2, Supporting Information). These results underscored that HIC1 expression was an independent prognostic factor, as it remains significantly associated with patient survival after accounting for these other factors. Next, we extracted mRNA and protein from a panel of eight clinical tissue specimens and experimental results showed that HIC1 was downregulated in GC tissues at both protein and mRNA levels (Figure 1E,F). These data revealed that HIC1 was downregulated and enhancement of HIC1 expression may be important for gastric cancer progression.

### 2.2 HIC1 Promotes Pyroptosis of GC Cells

**Figure 2:**
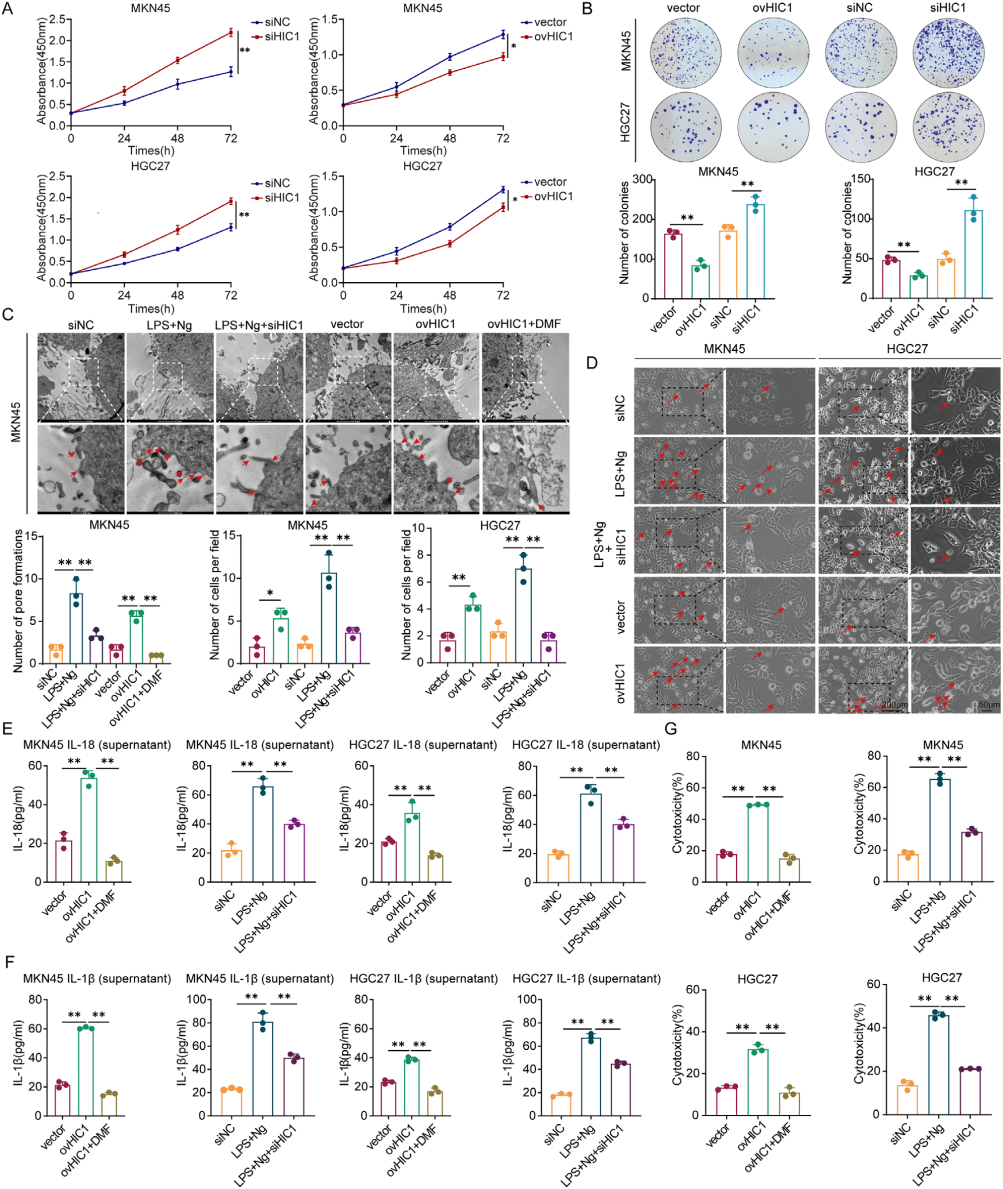
HIC1 facilitates the initiation of pyroptosis in GC cell lines. (A) CCK8 assays were employed to detect cell viability at the specified time points for 0, 24, 48, and 72 h. (B) Colony formation assays were conducted to evaluate the effects of HIC1 overexpression or knockdown, along with corresponding controls, on MKN45 and HGC27 cells. (C) Transmission electron microscope photographs across various groups. Overexpression of HIC1 increased the amount of pyroptotic cells, while DMF co‐treatment decreased the number in MKN45 cells. Besides, LPS+Ng‐induced pyroptotic cell death was represented in the siHIC1 group (red arrows showed pore formation). (D) Representative micrographs of pyroptotic cells in MKN45 and HGC27 cells across the aforementioned groups (red arrows indicated pyroptotic cells with enlarged bubbles). (E‐F) The levels of IL‐18 and IL‐1β secreted in the cell supernatant were quantified using ELISA kits. (G) LDH release assay of gastric cancer cells following HIC1 overexpression or siHIC1. *p < 0.05, **p < 0.01.

To evaluate the biological functions of HIC1 in GC cells, a CCK‐8 assay was conducted and results indicated that HIC1 overexpression led to a pronounced decrease in cell viability, while the knockdown of HIC1 promoted cell viability compared to control cells (**Figure** **2**A). Subsequently, colony formation assays were performed to probe into the proliferative capacity of gastric cancer cells. Notably, HIC1 overexpression significantly inhibited cell growth. In contrast, the effect was reversed upon HIC1 knockdown (Figure 2B). Next, to further investigate whether HIC1 was correlated with the mediation of pyroptotic events in GC progression, Lipopolysaccharide (LPS) was combined with Nigericin (Ng) to induce pyroptosis in GC cell lines. Through the utilization of transmission electron microscopy (TEM), we found that the number of pore formations experienced a considerable elevation following the administration of LPS plus Ng or HIC1 overexpression. However, the knockdown of HIC1 demonstrated remarkable resistance to this phenomenon in the LPS plus Ng group (Figure 2C,D). Furthermore, our examination revealed that the GSDMD inhibitor dimethylfumarate (DMF)^[^ ^20^ ^]^ resulted in a marked reduction in the proportion of pyroptotic cells induced by HIC1 overexpression (Figure 2C). The appropriate concentration of DMF (100 µm) was verified through western blot analysis conducted on MKN45 and HGC27 cells. (Figure S2A,B, Supporting Information). Identical findings were also acquired from the propidium iodide (PI) (Figure S1A,C, Supporting Information), Trypan blue (Figure S1B,D, Supporting Information) staining, and TUNEL assays (Figure S1E, Supporting Information), implying compromised cell membrane integrity. Upon disruption of cellular membrane integrity, lactate dehydrogenase (LDH) and proinflammatory cytokines such as IL‐18 and IL‐1β were released extracellularly. The levels of LDH and the content of IL‐18 and IL‐1β in the supernatant of MKN45 and HGC27 cells were determined, revealing a significant increase following HIC1 overexpression. (Figure 2E–G). Taken together, HIC1 overexpression contributed to pyroptosis, and HIC1 silencing impeded the progression.

### 2.3 HIC1 Regulates Cell Pyroptosis By Upregulating GSDMD

**Figure 3:**
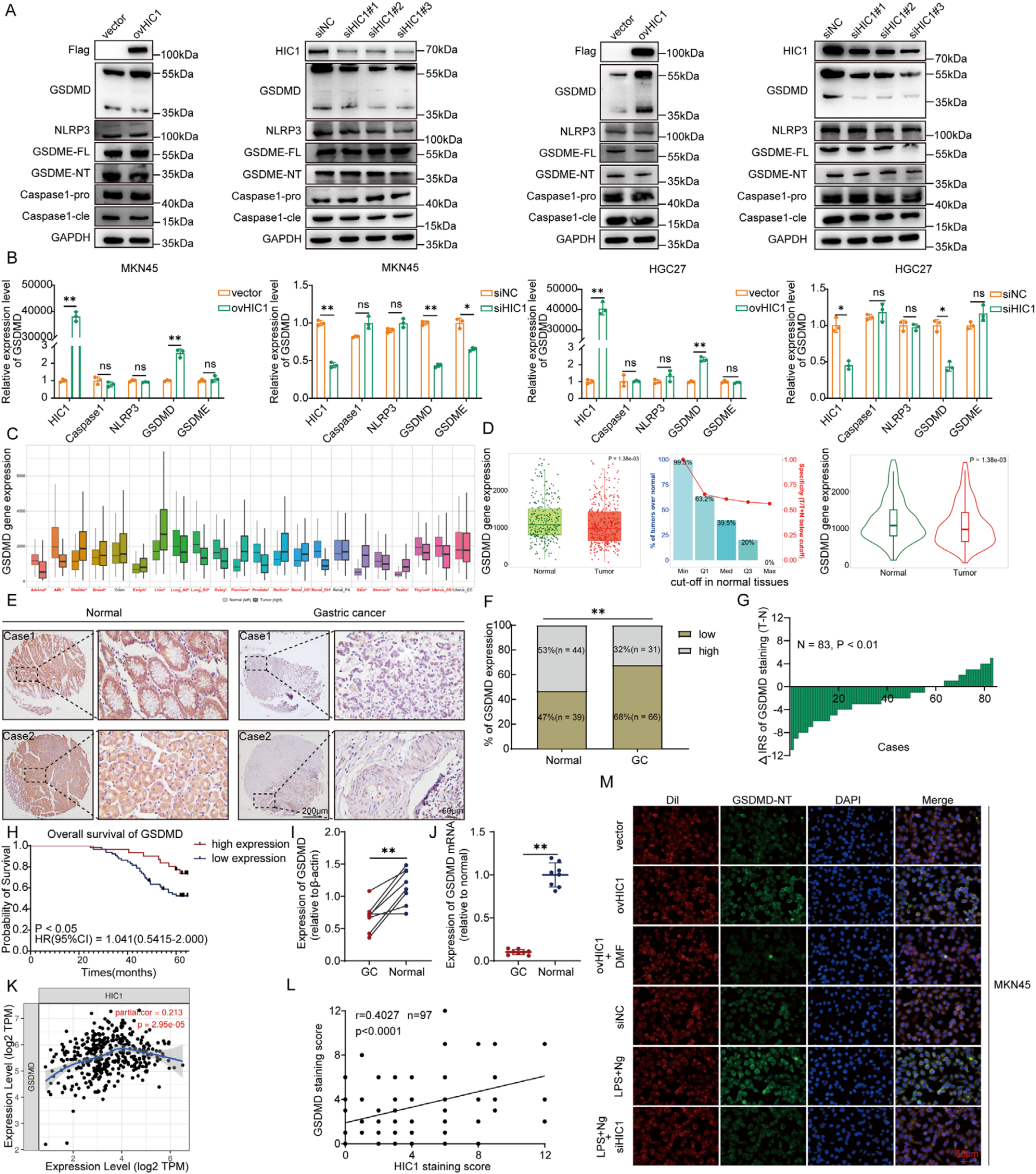
HIC1 promotes GSDMD expression and GSDMD is correlated with clinicopathological parameters. (A) Western blot analysis of protein levels of HIC1, NLRP3, Caspase‐1, GSDME, and GSDMD in the MKN45 (left) and HGC27 (right) cells. (B) The level of mRNA linked to pyroptosis was examined following HIC1 overexpression or depletion using RT‐qPCR analysis. (C,D) The GSDMD expression levels across multiple types of tumor and normal tissues were acquired from the TNMplot database (https://www.tnmplot.com/). (E) GSDMD immunochemical staining was performed on a tissue microarray (TMA). (F) Statistical analysis revealed that high expression of GSDMD in normal tissues and low expression in GC tissues. (G) The distribution of differences in GSDMD immunoreactivity scores (IRS) (∆IRS = IRS_T_−IRS_N_) was analyzed. (H) Kaplan‐Meier survival analysis was conducted to assess the impact of GSDMD expression on GC patient outcomes. (I,J) Quantitative evaluation of GSDMD expression at both protein and mRNA levels. (K) Correlation analysis between HIC1 and GSDMD was downloaded from the TIMER database. (L) Immunohistochemical staining demonstrated a strong positive correlation (*r* = 0.4027, *n* = 97, *p* < 0.0001) between the expression of HIC1 and GSDMD. (M) IF assays were utilized to determine the expression of GSDMD‐NT by observing alterations in fluorescence density. NS > 0.05, *p < 0.05, **p < 0.01.

To explore the impact of HIC1 on cell pyroptosis, we utilized the western blot and RT‐qPCR assays to determine the expression of proteins associated with pyroptosis. Our findings revealed that HIC1 overexpression triggered the upregulation of GSDMD gene expression, whereas HIC1 deletion resulted in a corresponding decrease. Nevertheless, the bands of NLRP3, Caspase‐1, and GSDME exhibited no discernible alterations in either protein or mRNA levels (**Figure** **3**A,B). To gain greater insight into understanding the important roles of GSDMD in the advancement of gastric cancer, TNMplot database described a substantial decrease in GSDMD expression levels in gastric cancer tissues compared to normal tissues. (Figure 3C,D). Subsequently, we performed IHC staining and observed the downregulation of GSDMD in 97 gastric cancer tissues (Figure 3E–G). Kaplan Meier analysis displayed that the lower expression of GSDMD was associated with a poorer prognosis (Figure 3H). Then, we further clarified the expression pattern of GSDMD in GC progression using eight tissue samples (Figure 1F; Figure 3I,J). Moreover, the correlation analysis between GSDMD and HIC1 was derived from the TIMER database, presenting a positive association consistent with the aforementioned observations (Figure 3K,L). And HIC1 was positively associated with GSDMD across multiple cancer types, including bladder cancer (BLCA), breast cancer (BRCA), lung squamous cell carcinoma (LUSC), pancreatic cancer (PAAD), and colon cancer (PARD) (Figure S3A–C, Supporting Information). These analyses provided a deeper understanding of the potential roles of HIC1 and GSDMD in different cancers and their underlying interactions. Afterward, immunofluorescence assays illustrated that HIC1 overexpression augmented the fluorescence intensity of GSDMD‐NT, which was attenuated upon co‐treatment with DMF inhibitor. On the contrary, the combination of LPS and Ng substantially enhanced its expression, with this effect being reversed upon HIC1 knockdown in MKN45 and HGC27 cells (Figure 3M; Figure S2C, Supporting Information). The preceding data were acquired under impermeabilized conditions. Following permeabilization, phalloidin staining was subjected to observe the morphology and the fluorescent alternation of GSDMD‐NT was visualized (Figure S2D, Supporting Information). In conclusion, HIC1 facilitates GSDMD expression.

### 2.4 HIC1 Transcriptionally Activates GSDMD Expression

**Figure 4:**
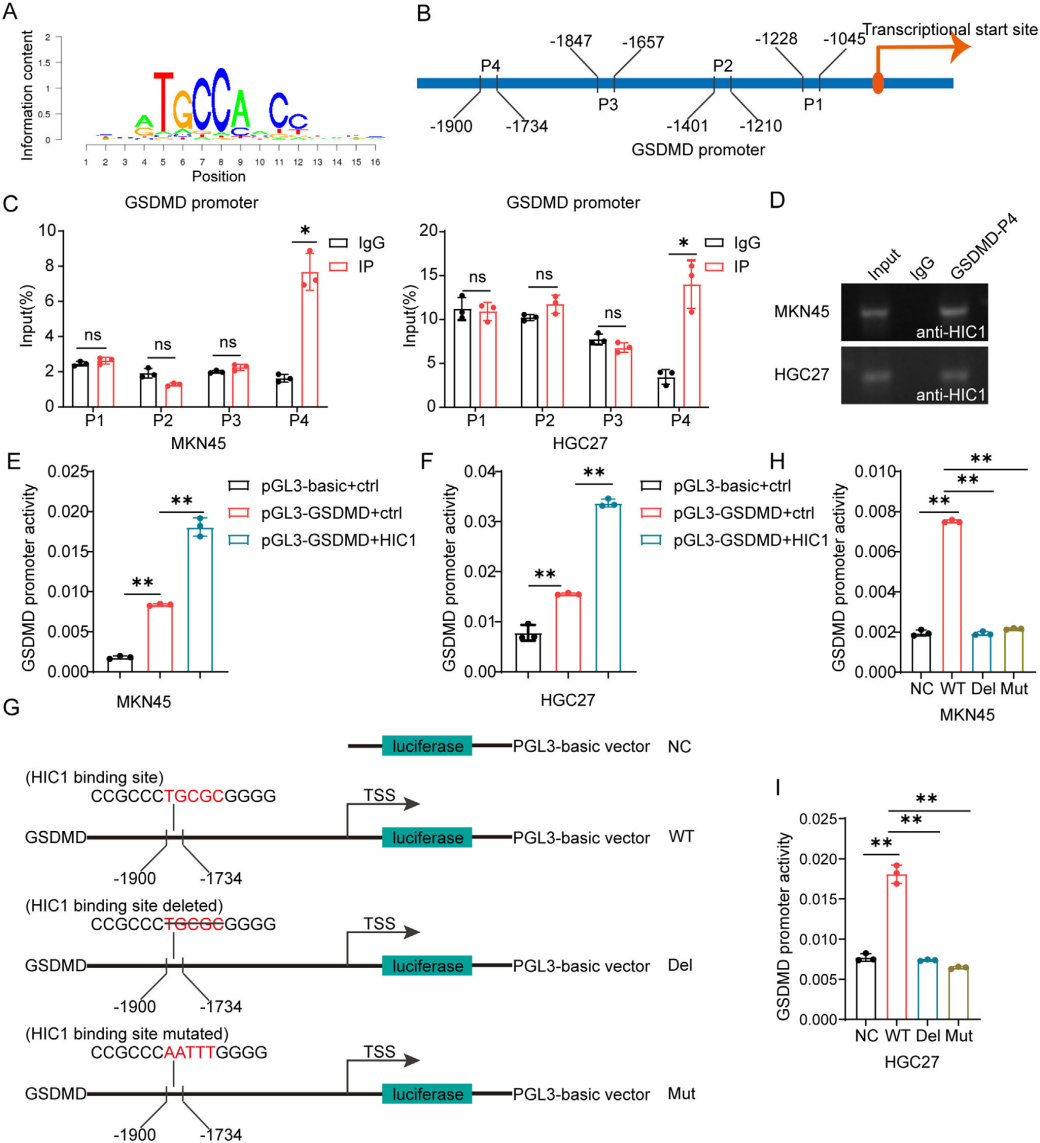
HIC1 binds to the promoter of GSDMD and activates GSDMD at the transcriptional level. (A) The Cistrome Data Browser (http://cistrpme.org/db) was applied to discover motif sequences of HIC1. (B) Putative binding sites in the promoter region of GSDMD were obtained from the hTFtarget (http://bioinfo.life.hust.edu.cn/hTFtarget). (C) ChIP assays confirmed the enrichment of HIC1 in the ‐1734/1900 fragments of the GSDMD promoter in both MKN45 and HGC27 cells. (D) The binding effectiveness was validated using agarose gel electrophoresis. (E,F) The pGL3‐basic and GSDMD promoter activity in MKN45 and HGC27 cells were assessed after co‐transfection with Renilla luciferase and pcDNA3.1‐FLAG‐HIC1 constructs. Control cells were transfected with the “empty” vector. (G) Schematic representation of PGL3‐basic (NC), wild type (WT), mutant (Mut), and deleted (Del) luciferase reporter gene constructs. (H,I) The activity of the WT, mutant, and deleted plasmids in GC cells co‐transfected with Renilla luciferase and a construct expressing HIC1. NS > 0.05, *p < 0.05, **p < 0.01.

In the prior investigations, we detected there was a noteworthy positive correlation between HIC1 and GSDMD gene expression. Consequently, we speculated that HIC1 activated GSDMD at the transcriptional level. To substantiate this, we initially identified the HIC1 motif (**Figure** **4**A) and discovered potential binding sites within the promoter region of GSDMD through the hTFtarget Database. Based on these, we designed four pairs of specific primers, comprising −1045/−1228, −1210/−1401, −1657/−1847, and −1734/−1900, using the 76‐kDa molecular weight of the HIC1 antibody (Santa Cruz) for DNA amplification by RT‐qPCR (Figure 4B). As anticipated, ChIP assays unequivocally validated that the −1734/−1900 region of the GSDMD promoter mediated its interaction with HIC1 in MKN45 and HGC27 cells (Figure 4C). Subsequent agarose gel electrophoresis assays were performed to authenticate the binding effectiveness and the results were in accordance with ChIP assays (Figure 4D). After that, we constructed GSDMD wild‐type (WT) promoter/reporter fusion constructs containing progressive 5′ deletions from ‐1734/‐1900, which comprised critical HIC1 binding sites. Our experimental findings demonstrated that the fusion constructs incorporating the full‐length GSDMD promoter exhibited a more than four‐fold increase in activity compared to the control group. Moreover, the co‐transfection of HIC1 further activated GSDMD promoter activity (Figure 4E,F). Concurrently, PGL3‐basic (NC), GSDMD deleted (Del), and mutated (Mut) constructs were designed for dual luciferase promoter reporter assays (Figure 4G). Nevertheless, in contrast to the wild type, the activity of the deletion or mutation constructs decreased, respectively, signifying the abolishment of HIC1‐mediated activation functions (Figure 4H). Consistently, analogous results were also noted in HGC27 cells (Figure 4I). Collectively, HIC1 protein binds to the GSDMD promoter, thereby stimulating its expression.

### 2.5 Jab1 Directly Interacts with HIC1 to Mediate Ubiquitylation at Lysine 517

**Figure 5:**
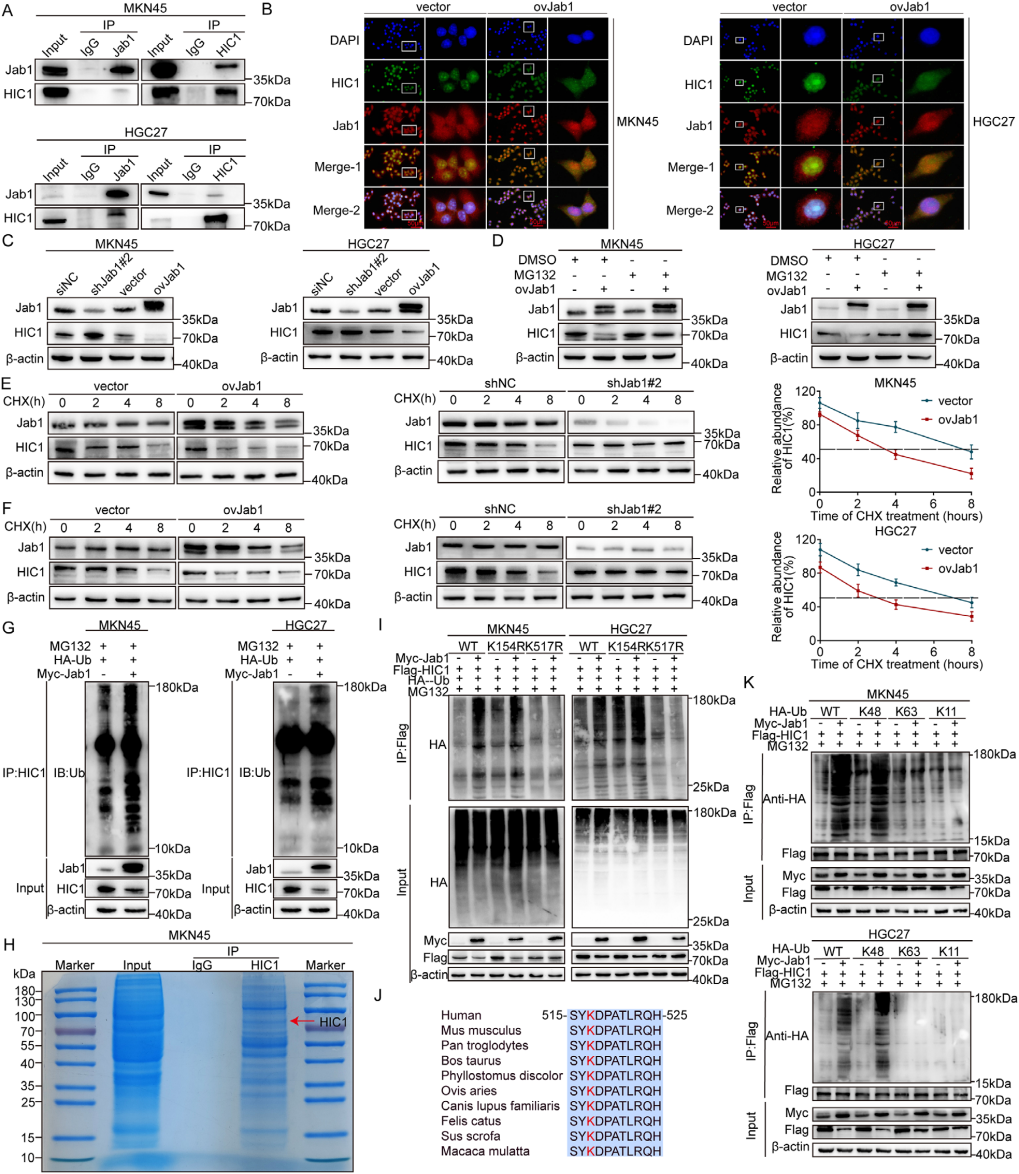
Jab1 specifically interacts with HIC1 and mediates the degradation of HIC1 in a ubiquitin‐proteasome manner at K517. (A) Whole‐cell extracts of MKN45 and HGC27 cells were isolated and subjected to a Co‐IP assay to investigate the interaction between endogenous Jab1 and HIC1. (B) IF assay was employed to detect the co‐localization of HIC1 and Jab1. (C) Western blot analysis revealed a significant reduction in HIC1 protein levels following transfection with Myc‐Jab1 overexpression plasmid. (D) Cell lysates were collected for electrophoresis following treatment with MG132 (20 µm) for 6 h. (E,F) Western blot was subjected to explore the impact of Jab1 overexpression on the protein stability of HIC1 for MKN45 and HGC27 cells, as compared to an empty vector. CHX (50 µm). (G) Myc‐Jab1, Flag‐HIC1, and HA‐ubiquitin plasmids were simultaneously transfected into cells and immunoprecipitated using HIC1 antibody prior to being immunoblotted. (H) Coomassie blue staining of the HIC1 protein complex with red arrows indicating the bands corresponding to HIC1 (76 kDa). (I) Ubiquitylation assays were carried out with FLAG‐tagged wild‐type HIC1 or lysine‐to‐arginine mutants K154R and K517R together with HA‐Ub plasmid. (J) Alignment of the region corresponding to amino acids 515–525 of human HIC1 across different species. (K) GC cells transfected with the indicated plasmids were subjected to a ubiquitination experiment with the indicated antibodies.

To shed light on the molecular mechanisms underlying the downregulation of HIC1 in GC, we performed mass spectrometry analysis and coimmunoprecipitation assay to identify HIC1‐interacting proteins (Figure S4A, Supporting Information). Screening of several proteins expressed in MKN45 cells verified that Jab1 specifically interacted with HIC1 (**Figure** **5**A). Furthermore, immunofluorescence experiments were conducted to uncover their intracellular co‐localization of endogenous HIC1 and Jab1 in MKN45 and HGC27 cells. The result discovered that HIC1 was predominantly located in the nucleus, whereas Jab1 was observed in both the nucleus and cytosol without any intervention. Interestingly, HIC1 increased the percentage of in cytoplasm and decreased the nucleus after Jab1 overexpression, indicating that Jab1 facilitated HIC1 protein entry into the cytoplasm (Figure 5B). Following that, we evaluated the expression profiles of Jab1 in GC tissues through TIMER database as well as TNMplot website. The result disposed a significant upregulation of Jab1 expression in GC (Figure S4B,C, Supporting Information). Consistent findings were obtained in 6 paired GC tissues and the adjacent normal counterparts by western blot and RT‐qPCR assays (Figure S4D, Supporting Information). To avoid the possibility of off‐target effects caused by a single sequence, we employed two distinct shJab1 sequences and, based on the experimental results, selected shJab1#2 for use in subsequent experiments (Figure S4E, Supporting Information). In line with the aforementioned hypothesis, Jab1 knockdown apparently elevated the expression level of HIC1 in GC cells. Conversely, overexpression of Jab1 resulted in a lower HIC1 protein level compared to the negative control (Figure 5C). Then additionally, the endogenous HIC1 markedly reduced upon transfection with accumulating doses of Jab1 in MKN45 and HGC27 cells (Figure S4F, Supporting Information). Briefly, Jab1 was negatively related to HIC1 at the protein level. Importantly, HIC1 downregulation mediated by Jab1 knockdown was restored following treatment with the proteasome inhibitor MG132, indicating that Jab1 promoted HIC1 degradation through the proteasome pathway (Figure 5D). To determine whether Jab1 regulated HIC1 abundance at the post‐translational level, we performed a half‐life assay with cycloheximide (CHX) to block protein synthesis. The findings suggested that overexpression of Jab1 significantly accelerated the protein degradation, resulting in a shortened half‐life, whereas Jab1 knockdown had the opposite effect in MKN45 and HGC27 cells (Figure 5E,F; Figure S4G, Supporting Information). Subsequently, we accessed the impact of Jab1 on HIC1 ubiquitination. The results displayed that Jab1 overexpression enhanced the level of ubiquitination in GC cells (Figure 5G). An in vitro ubiquitination assay was also conducted, revealing that the presence of Jab1 protein significantly facilitated the ubiquitination of HIC1 in vitro (Figure S4H, Supporting Information). Overall, these observations underscored the role of Jab1 in promoting the degradation of HIC1 via the ubiquitin‐proteasome pathway.

To gain a deeper insight into the regulatory mechanism for HIC1 ubiquitination, we performed Coomassie blue staining (Figure 5H), silver staining (Figure S4I, Supporting Information), and mass spectrometry analysis to pinpoint the precise ubiquitinated sites. Our results revealed that ubiquitination sites were chiefly concentrated on lysine residues K154 and K517 (Figure S4J, Supporting Information). We next, respectively, generated specific plasmids in which lysine residue was substituted with arginine at position 154(K154R) and 517(K517R). Evidently, HIC1(K154R) exhibited a pronounced ability to promote extensive ubiquitylation. However, overexpression of Jab1 had no distant effect on HIC1 ubiquitination in the K517R mutant, suggesting that K517 was required for Jab1‐mediated ubiquitination (Figure 5I). And the sequences were highly conserved from humans to macaca mulatta (Figure 5J). In addition, we used specific antibodies against K48‐linked, K11‐linked, or K63‐linked polyubiquitin chains to identify the type of ubiquitin chains responsible for HIC1 degradation. The results confirmed that Jab1 specifically facilitated the polyubiquitination of HIC1 through K48 linkage, while no significant effects were observed on K11‐ or K63‐linked polyubiquitination of HIC1(Figure 5K). These findings, in conjunction with the data, indicated that Jab1 targets HIC1 for ubiquitylation at K517.

### 2.6 HIC1 Inhibits Tumorigenicity In Vivo and Enhances the Efficacy of PD‐L1 Therapy

**Figure 6:**
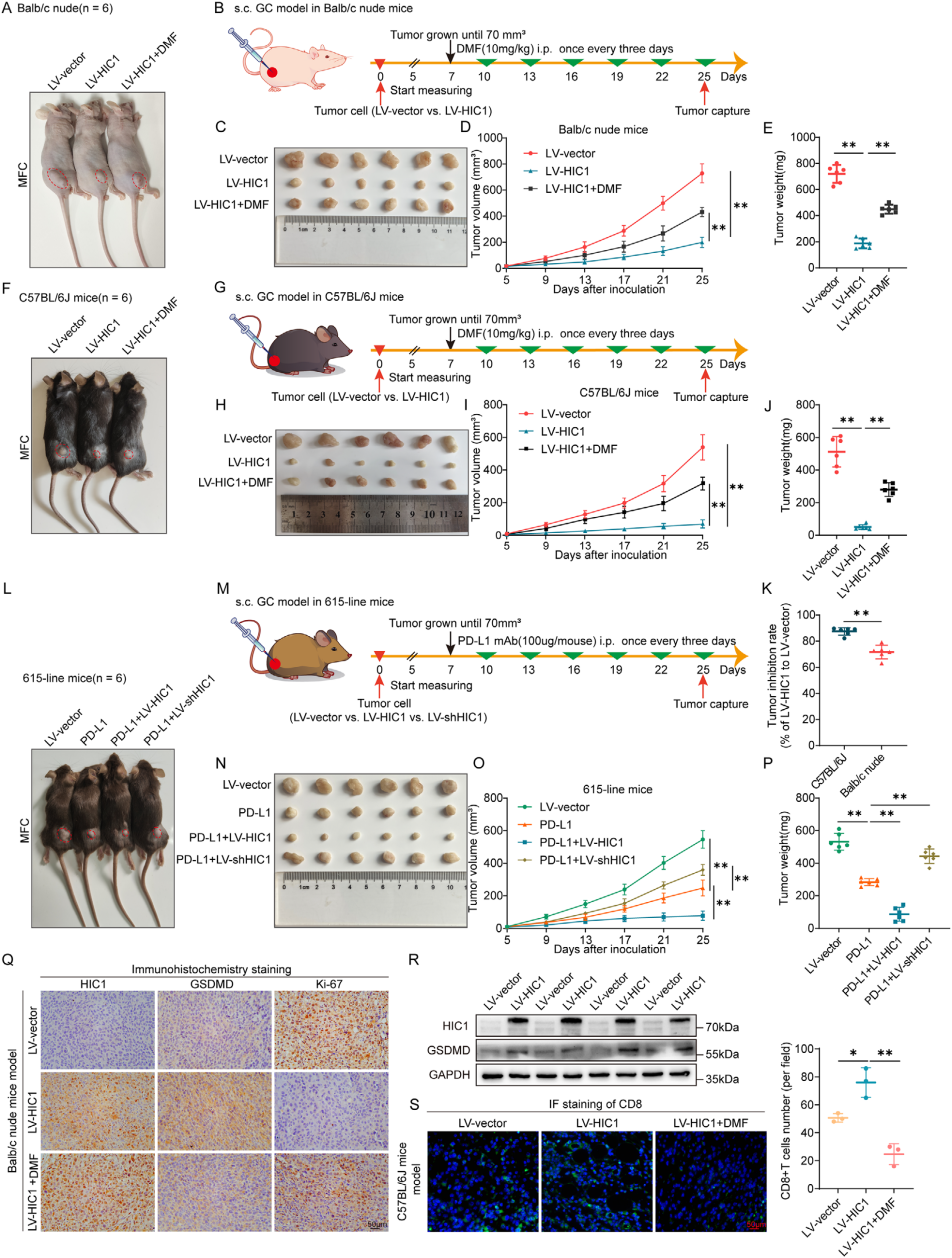
HIC1 suppresses tumor growth in vivo and synergies with PD‐L1 antibodies in functioning anti‐tumor effects. (A–C) MFC cells treated with LV‐vector and LV‐HIC1 were transplanted into BALB/C nude mice (*n* = 6). DMF (10 mg kg^−1^) was administered intraperitoneally once the tumor volume reached 70 mm^3^. Tumors were collected and photographed at the termination of the trial. (D,E) Tumor volumes and weights of the indicated group. (F) Representative images of C57BL/6J mice model bearing MFC cells. (G) Illustration depicting the protocol for DMF administration in the subcutaneous GC model. (H) Xenograft tumors from C57BL/6J mice. (I,J) Overexpression of HIC1 inhibited tumor growth, while DMF co‐treatment rescued the subcutaneous tumor size reduced by LV‐HIC1 in MFC cells, as compared to the negative control. (K) Display of the inhibition rate in BALB/C nude and C57BL/6J mice. (L–N) Transplantation of MFC cells into 615‐line mice followed by PD‐L1 antibody treatment (100 µg per mouse). (O,P) Evaluation of tumor volumes and weights. (Q) IHC staining for HIC1, GSDMD, and Ki‐67 in tumor tissues from BALB/C nude mice. (R) Western blot analysis of HIC1 and GSDMD expression in tumors. (S) Representative IF staining and quantification analysis of CD8 cells in tumor tissues for LV‐vector, LV‐HIC1, and LV‐HIC1+DMF groups. *p < 0.05, **p < 0.01.

To clarify the role of HIC1 in GC cell growth and antitumor immunity responses, subcutaneous xenograft GC models were established by inoculating MFC cells with lentivirus encoding HIC1 into the right hips of BALB/C nude mice (immunodeficient), followed by treatment with GSDMD inhibitor DMF (10mg kg^−1^) until the tumor volumes reached 70 mm^3^ (**Figure** **6**A–C). The results revealed that the HIC1 overexpression group led to a marked reduction in tumor volumes and weights compared with the negative control group, while the DMF co‐treated group experienced a noticeable increase (Figure 6D,E). Subsequently, we implanted MFC cells with HIC1 overexpression into C57BL/6J mice (immunocompetent) (Figure 6F–H) and found that HIC1 overexpression exhibited a stronger inhibition impact on tumor volumes and weights than controls, as well as BALB/C nude mice. The group co‐administered with DMF consisted of BALB/C nude mice, namely increased the tumor growth significantly but less than the controls (Figure 6I,J). It is worth noting that overexpression of HIC1 resulted in a higher tumor inhibition rate of ≈80% in C57BL/6J mice, however, it only trigged a tumor inhibition rate of ≈60% in BALB/C nude mice (Figure 6K), suggesting the tumor suppression effect of HIC1 was partially reliant on an efficient immune system. Furthermore, to further elucidate the impact of PD‐L1 antibody on the therapeutic efficacy of the combined treatment involving LV‐HIC1 or LV‐shHIC1, MFC‐challenged subcutaneous GC model in 615‐line mice (immunocompromised) was constructed. Once the tumor volumes had grown 70 mm^3^, the 615‐line mice were administrated with PD‐L1 (100 µg per mouse) antibody through intraperitoneal injections once every three days (Figure 6L–N). The PD‐L1 antibody group partially impeded tumor progression, and the synergistic effect of HIC1 overexpression combined with PD‐L1 antibody further augmented the anti‐tumor response. Conversely, treatment with shHIC1 in combination with PD‐L1 antibody revealed an obvious increase in tumor growth compared to the PD‐L1 group (Figure 6O,P). To probe into the association between HIC1 expression and immunotherapeutic responses, we utilized the ROC Plotter database, which included data on gastric cancer patients treated with immunotherapy, specifically anti‐PD‐1(pembrolizumab) agents. In agreement with existing literature,^[^ ^21^ ^]^ our analysis also revealed that HIC1 expression was significantly elevated in patients who did not respond to immunotherapy (Figure S5A, Supporting Information). Besides, we carried out additional experiments to verify the synergistic effect of Jab1 knockdown and immune checkpoint inhibitor therapy (Figure S5B, Supporting Information). The results indicated that compared to the control group, either Jab1 blockade or PD‐L1 treatment alone significantly reduced tumor volume and weight. Notably, the combination of Jab1 blockade and PD‐L1 resulted in a more pronounced tumor suppression effect, leading to a greater reduction in tumor size (Figure S5C–E, Supporting Information). Eventually, transplanted tumors were harvested for a detailed evaluation of HIC1 expression. Using immunochemical staining, the subcutaneous xenograft GC mice with HIC1 overexpression demonstrated elevated levels of GSDMD expression and decreased proliferation, as indicated by Ki‐67 staining (Figure 6Q). In line with this, western blot analysis of the HIC1 overexpression group also exhibited higher GSDMD expression than the control group (Figure 6R). Additionally, IF assays found that HIC1 overexpression augmented the amount of CD8 cell infiltration in GC mouse tissues (Figure 6S). Collectively, these findings suggested that targeting HIC1 potentiated the therapeutic efficacy of PD‐L1 therapy in GC.

### 2.7 High Expression of HIC1 Enhances CD8^+^ T Cells Infiltration

**Figure 7:**
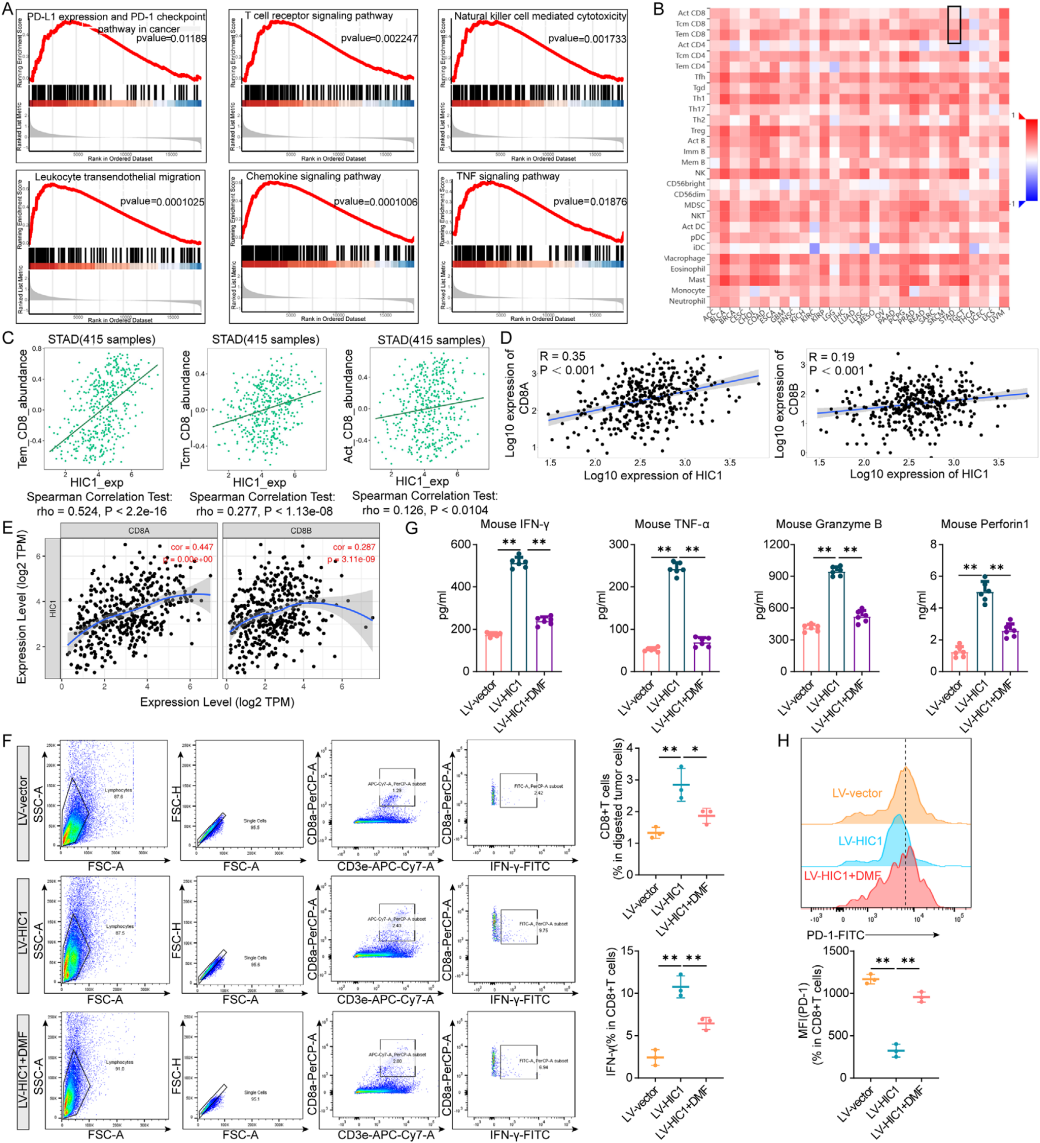
High HIC1 expression is positively associated with the infiltration of CD8^+^ T cells. (A) GSEA analysis of HIC1‐related differential pathways. (B,C) Utilizing the TISIDB database, correlations between HIC1 and various immune cells in gastric cancer, including Tem, Tcm, and Act CD8, were identified. (D‐E) TNMplot and TIMER databases displayed the relationships between HIC1 expression and CD8A and CD8B mRNA in gastric cancer. (F) Flow cytometry was performed to calculate the proportion of CD8^+^ T cells and IFN‐γ sorted from CD8^+^ T cells that had infiltrated the tumors of the specified groups. (G) The contents of IFN‐γ, TNF‐α, Granzyme B, and perforin 1 in C57BL/6J mouse tumors were measured via the ELISA Mouse Kit. (H) The medium fluorescence intensity (MFI) of PD‐1 in a total of CD8^+^ T cell populations was depicted. *p < 0.05, **p < 0.01.

To investigate whether HIC1 participated in the immune cell infiltrations, gene‐set enrichment analysis (GSEA) was utilized to visualize the essential enrichment pathways of HIC1. Bioinformatics database demonstrated that HIC1 engaged in the PD‐L1 expression and PD‐1 checkpoint pathway in cancer, T cell receptor signaling pathway, Natural killer cell mediated cytotoxicity, Leukocyte transendothelial migration, Chemokine signaling pathway and TNF signaling pathway, which were closely related with immune regulation (**Figure** **7**A). Studies presented that CD8^+^ T cells typically played an essential role in combating tumors and elevated expression of CD8^+^ T cells predicted a favorable prognosis in treating patients.^[^ ^22^ ^]^ Therefore, we utilized TISIDB website to screen the relationship between HIC1 and immune cell infiltrations in a majority of cancers, which emphatically shed light on the correlation between CD8^+^ T cells and HIC1 in gastric cancer. As shown in Figure 7B,C, HIC1 was strongly positively associated with CD8^+^ T cell subsets (Tem, Tcm, and Act CD8). Additionally, similar results were also derived from TNMplot and TIMER databases (Figure 7D,E). To further corroborate the bioinformatics results, we employed immunohistochemistry to observe the relationship in 48 clinical gastric cancer samples. Results unveiled that CD8A expression was significantly higher in normal tissues compared to gastric cancer tissues and exhibited a strong correlation with HIC1 (Figure S6, Supporting Information).

Through extensive database analysis, we also have uncovered a robust association between GSDMD expression and T cell infiltration, particularly CD8^+^ T cells (Figure S7A–C, Supporting Information). Furthermore, GSDMD was strongly related to CD8A and CD8B (Figure S7D,E, Supporting Information). These results suggested that GSDMD may play a crucial role in immune responses, especially in the recruitment and activation of CD8^+^ T cells. Then, we carried out an ELISA assay to assess whether GSDMD mediated the functional role of HIC1 in regulating pyroptosis. The findings pointed out that overexpression of HIC1 significantly promoted the secretion of key pro‐inflammatory cytokines, such as IL‐18 and IL‐1β. However, when GSDMD was silenced, this enhancement of secretion induced by HIC1 was effectively reversed (Figure S7F, Supporting Information). To summarize, we concluded that GSDMD has a non‐negligible role in HIC1‐mediated immune responses and pyroptosis.

In parallel, we evaluated the interaction between Jab1 and cytotoxic T cells. GO and GSEA stated that Jab1 was implicated in key biological processes in gastric cancer, including the regulation of adaptive immune responses and leukocyte activation (Figure S8A,B, Supporting Information). Data further underscored that Jab1 expression was negatively correlated with the abundance of Tem and Tcm CD8 cells, a finding that was corroborated using the TIMER database (Figure S8C–G, Supporting Information). On the other hand, we delved into the role of Jab1 in the regulation of pyroptosis using optical microscopy. Our observations indicated that the number of pyroptotic cells in both the shJab1#1 and shJab1#2 groups was significantly higher than in the control group, with shJab1#2 exhibiting a more pronounced effect. Notably, the knockdown of HIC1 was able to rescue the pyroptotic phenotype induced by Jab1 depletion (Figure S9A, Supporting Information). Besides, ELISA assays were consistent with the aforementioned findings (Figure S9B, Supporting Information). Based on these data, we considered that Jab1 played a critical role in the regulation of pyroptosis and immunity.

**Figure 8:**
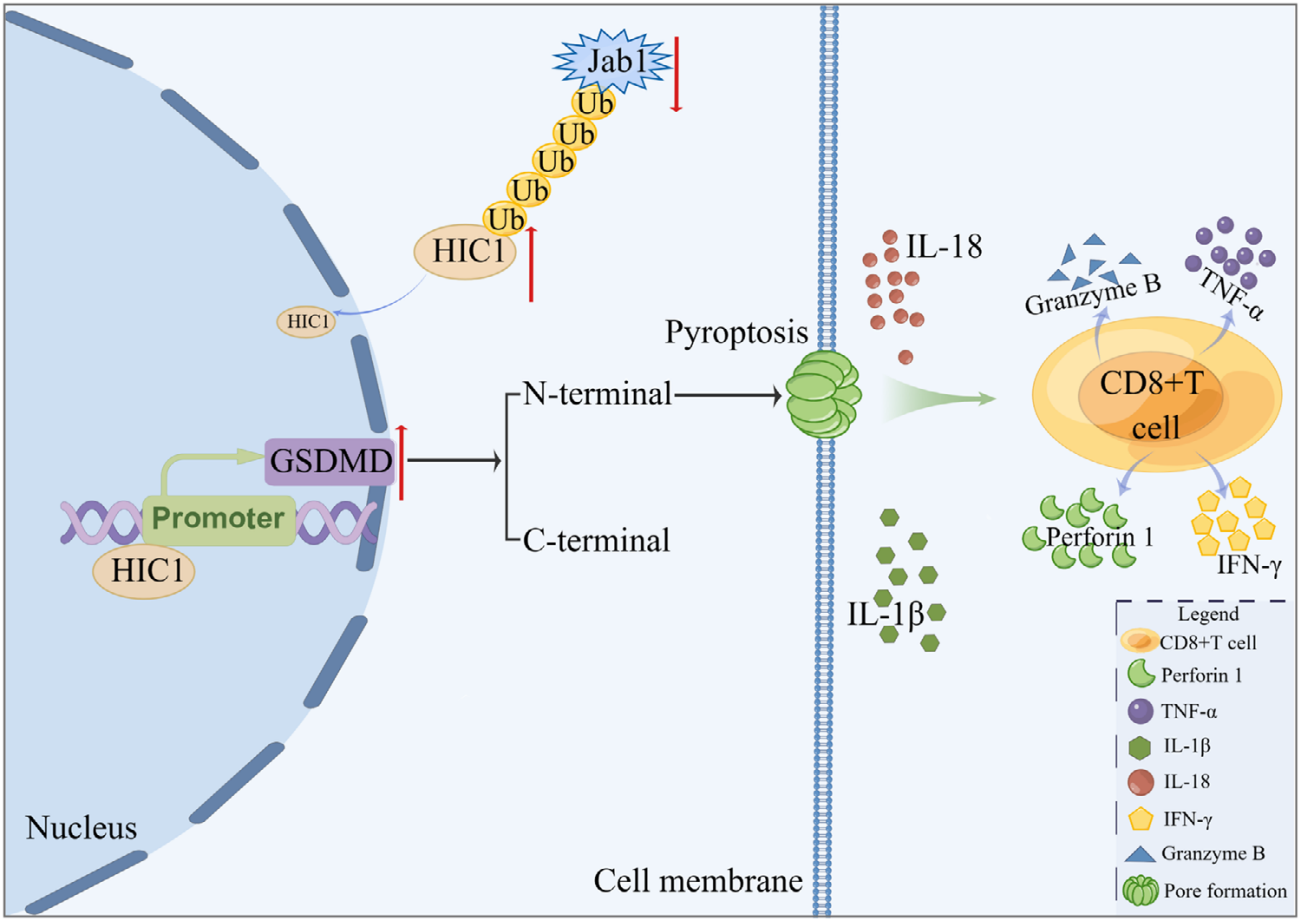
The schematic diagram illustrated that HIC1 impeded GC progression via activation GSDMD expression and augmentation CD8^+^ T cell infiltration (By Figdraw).

Thereafter, we quantified the population of CD8^+^ T cells and isolated IFN‐γ from CD8^+^ T cells within tumor tissues by establishing a C57BL/6J mice model. Our findings displayed that upregulation of HIC1 augmented the infiltrative percentage of CD8^+^ T cells and IFN‐γ. Interestingly, this effect was largely reversed under co‐treatment with DMF conditions (Figure 7F). ELISA Mouse Kit was applied to measure the secretion of cytokines including IFN‐γ, TNF‐α, Granzyme B, and Perforin 1. Our findings displayed tumors in the LV‐HIC1 group had higher amounts of IFN‐γ, TNF‐α, Granzyme B, and Perforin 1 compared to those in the control group. However, concomitant treatment of DMF with HIC1 exerted the opposite effects (Figure 7G). In addition, flow cytometry was performed to monitor the mean fluorescent value of PD‐1 in CD8^+^ T cells (Figure 7H). These data corroborated that HIC1 modulated immune responses by recruiting CD8^+^ T cells. Eventually, a graphical representation illustrated that HIC1 boosted CD8^+^ T cell infiltration by increasing GSDMD expression (**Figure** **8**).

## 3 Discussion

The tumor suppressor HIC1 is widely recognized for its involvement in regulating cellular growth, cell metastasis, and cell cycle across a broad spectrum of human cancers.^[^ ^15^, ^23^ ^]^ However, the potential functions and molecular mechanisms of HIC1 in cancer immunity and immunotherapies remain incompletely investigated. From the GSEA analysis for screening HIC1‐related pathways, we presented compelling evidence that HIC1 was positively associated with immune responses in GC. It has been documented that CD8^+^ T cells are critical for cancer immunosurveillance by secreting cytotoxic cytokines, including Granzyme B and IFN‐γ.^[^ ^24^ ^]^ Consequently, our primary focus centered on elucidating the correlation between HIC1 and CD8^+^ T cells, which was corroborated by bioinformatics databases, confirming a robust positive association. Based on these, our findings demonstrated that HIC1 overexpression enhances the amount of CD8^+^ T cells and IFN‐γ. Notably, HIC1 augmented the level of PD‐1 in a total of CD8^+^ T cells, thereby improving the cytotoxic T‐cell killing efficacy. This raises a critical question regarding the mechanism of action of HIC1 within the immune system and necessitates further exploration into potential interconnected pathways.

More recently, emerging studies have described the critical role of pyroptosis in tumor suppression, attributed to the identification of epigenetic silencing and genetic mutations in certain gasdermins (GSDMs) among cancer patients.^[^ ^20^ ^]^ It has been proposed that GSDMB‐ and GSDME‐dependent cell pyroptosis acted as an immunostimulating agent, curbing tumor advancement through the activation of lymphocyte cells and reshaping the immune landscape.^[^ ^25^ ^]^ Consequently, we hypothesized that HIC1 might increase CD8^+^ T cell infiltration into tumors by regulating pyroptosis. In vivo experiments displayed that overexpression of HIC1 impeded tumor growth in BALB/C nude and C57BL/6J mice models. The co‐treatment of HIC1 with the GSDMD inhibitor DMF led to an augmentation in both tumor volume and weight. Moreover, HIC1 synergized with PD‐L1 to suppress tumorigenesis in 615‐line mice models, indicating that elevated HIC1 expression may render tumor cells more responsive to PD‐L1 therapy. Furthermore, the reduction in tumor size can be attributed to the enhancement of CD8^+^ T cells, partially facilitated by the upregulation of HIC1 expression. Overall, the aforementioned findings underscored the potential of HIC1 as a promising immunotherapeutic biomarker.

As previous literature mentioned, the occurrence of pyroptosis involves a complex interplay of multiple proteins, including NLRP3, Caspase‐1, GSDMD, and GSDME, which play indispensable roles in shielding the host from perilous signals.^[^ ^26^ ^]^ Subsequently, we delved into the mechanisms through which HIC1 influenced pyroptosis in GC cells. Western blot and RT‐qPCR assays were subjected to screen for proteins associated with pyroptosis. Pyroptotic effector GSDMD emerged as a viable downstream target of HIC1. Subsequent findings indicated that HIC1 triggered the transcriptional activation of GSDMD. While it has been widely acknowledged that HIC1 is predominantly recognized as a transcriptional repressor, our current study posits that HIC1 may function as a transcriptional activator in driving gene expression in gastric cancer. To date, a few transcription factors, such as Snail,^[^ ^27^ ^]^ MEF2,^[^ ^28^ ^]^ nuclear receptors,^[^ ^29^ ^]^ have demonstrated a dual capacity in fostering gene expression. Therefore, it is justifiable to infer that HIC1 exhibits dual functionality as both a repressor and an activator through its interactions with diverse target genes to orchestrate cellular physiological processes.

In this study, we demonstrated that HIC1 was markedly downregulated in GC. As we all know, promoter hypermethylation frequently leads to the silencing of HIC1 in human cancers.^[^ ^15^, ^16^ ^]^ Following this, we postulated that alternative mechanisms may contribute to the downregulation of HIC1 expression in gastric cancer. Our observations uncovered that Jab1 facilitated the degradation of HIC1 via the ubiquitin‐proteasome pathway at K517. The correlation between HIC1 and Jab1 likely casts a new light on the potential mechanism underlying the downregulated expression of HIC1 in gastric cancer. However, further experiments are required to explore the cause of HIC1 nuclear translocation, which will provide critical and innovative perspectives on the biological implications of ubiquitination regulation.

Gastric cancer remains one of the leading causes of cancer‐related mortality globally, with a notably higher incidence in Asia. Currently, therapeutic approaches such as surgery, chemotherapy, radiotherapy, immunotherapy, and targeted therapy have demonstrated efficacy in the management of GC.^[^ ^1^, ^30^ ^]^ However, despite notable advancements in treatment strategies, several challenges persist. These include difficulties in early detection, tumor heterogeneity, therapeutic resistance, as well as recurrence and metastasis, which continue to hinder optimal patient outcomes.^[^ ^31^ ^]^ Immunotherapy, as an emerging treatment modality, has yielded promising results in select patients.^[^ ^7^ ^]^ As such, ongoing research and innovation are critical to developing more effective therapeutic options. Herein, we not only highlighted the pivotal role of HIC1 in recruiting the CD8^+^ T cells to infiltrate the tumor but also its synergy with PD‐L1 antibodies in enhancing antitumor immune responses. Although we did not delve deeply into the specific mechanisms, this study distinguishes itself from previous research by uncovering the potential role of HIC1 in the gastric cancer immune microenvironment, particularly within the context of the intricate interplay between pyroptosis and immune evasion. In conclusion, our findings demonstrated the innovative role of HIC1 as a transcriptional activator in GC, driving the initiation of pyroptosis and enhancing CD8^+^ T cell infiltration, which has certain novelty and creative significance. Studies have investigated that HIC1 participates in the regulation of pyroptosis to some extent through the promotion of oxidative stress.^[^ ^32^ ^]^ Further research should be undertaken in the future. Building upon the foundations of the current study, we aim to examine the involvement of HIC1 in other forms of cell death, such as apoptosis, necrosis, and ferroptosis, with the goal of gaining a more nuanced and comprehensive understanding of HIC1's role in pyroptosis and its broader implications in various biological processes. Collectively, targeting HIC1 would present an appealing immunotherapeutic strategy to improve outcomes in GC patients.

## 4 Experimental Section

### Clinical Samples

The human GC tissue microarray contained 83 normal gastric mucosa and 97 GC tissues. A total of eight tissues were obtained from patients who underwent gastric cancer surgery without any pre‐operative chemotherapy or radiotherapy. They were collected from the Affiliated Hospital of Xuzhou Medical University and stored in liquid nitrogen for the following experiments. All experiments were approved by the Ethics Committee of Affiliated Hospital of Xuzhou Medical University and each patient signed an informed consent form.

### Cell Culture

Human GC cell lines, MKN45 and HGC27, were preserved by our laboratory, and the mouse GC cell line MFC was derived from the Shanghai Institute of Biochemistry and Cell Biology, Chinese Academy of Sciences (Shanghai, China). They were maintained in Dulbecco's Modified Eagle's medium (DMEM, Gibco, Shanghai, China) supplemented with 10% fetal bovine serum (FBS, Gibco, Carlsbad, USA) in an incubator consisting of 5% CO_2_ at 37 °C.

### Gene Transfection and Silencing

HIC1 expression plasmids (pcDNA3.1‐HIC1) purchased from MiaoLing Biology (Wuhan, China) were applied for transient transfection in GC cells using Lipofectamine 2000 (Invitrogen, Carlsbad, CA, USA). Myc‐tagged human JAB1 overexpression plasmids or vector plasmids were derived from Shhebio (Shanghai, China). The sequence of siHIC1#3 (5′‐AGTTCGCACAGCAACGCAACCTCAT‐3′) and siNC (5′‐TTCTCCGAACGTGTCACGT‐3′) were synthesized by IBSBIO (Shanghai, China) and using jetPRIME (Polyplus Transfection, catalog: EF0651) for following experiment according to the manufacturer's instructions.

To obtain stable strains, the lentiviruses targeting HIC1 and corresponding empty vectors were designed, processed, and synthesized by OBiO Technology (Shanghai, China). Similarly, short hairpin RNA of human Jab1 (shJab1#2) was also inserted into lentiviral vectors and the sequences were listed: shJab1: 5′‐CCAGACTATTCCACTTAATAA‐3′; shNC: 5′‐TTCTCCGAACGTGTCACGT‐3′. Lentivirus vectors were added into MKN45 and HGC27 cells. After 6 h, the medium was replaced with a fresh complete medium including polybrene (10 µg mL^−1^) for 48 h. Ultimately, the cells were selected by 1 µg mL^−1^ puromycin for one week.

### Western Blot Analysis

Protein samples were extracted with RIPA lysis buffer containing proteinase inhibitor cocktail (KeyGEN BioTECH, catalog: KGP702) after cells were washed. Subsequently, the BCA Protein Assay Kit (Beyotime, Shanghai, China) was applied for quantification. Identical amounts of protein were separated by 10% SDS‐PAGE for electrophoresis and then transferred onto polyvinylidene fluoride membranes. After being blocked with 5% non‐fat milk for 2 h, the specific primary antibodies were incubated at 4 °C overnight for HIC1(1:1000 dilution, Santa Cruz), GSDMD (1:5000 dilution, ABclonal), NLRP3 (1:1000 dilution, ABclonal), Caspase‐1 (1:3000 dilution, ABclonal), GSDME (1:5000 dilution, ABclonal), Jab1(1:1000 dilution, Santa Cruz), USP8(1:1000 dilution, ABclonal), TRAF6 (1:1000 dilution, ABclonal), FBXL7 (1:1000 dilution, Abcam), followed by the HRP‐conjugated secondary antibodies incubation for ≈1 h at room temperature. At last, the TanonTM High‐sig ECL Western Blot Substrate (Tanon) was used to detect protein bands. Here, GAPDH and β‐actin (1:5000 dilution, Proteintech) were taken as internal controls.

### RNA Isolation and Real‐Time Quantitative Reverse Transcription PCR (RT‐qPCR)

Total RNA was harvested from cell lysates by TRIzol reagent (Invitrogen, Carlsbad, CA, USA) and transcribed into cDNA using SweScript RT I First Strand cDNA Synthesis Kit (Servicebio, Wuhan, China) according to the manufacturer's instructions. Following this, RT‐qPCR was conducted with SYBR Green qPCR Master Mix (Servicebio) to determine the expression of target RNAs. The sequences of the primers were normalized by GAPDH level or 18S level and were as follows: HIC1: Forward: 5′‐ TGACTTTTCCTGAAGCGGACA ‐3′, Reverse: 5′‐TGTCATGGTCCAGGTTGAGC‐3′; GSDMD: Forward: 5′‐CAGAAGGGACGTGGTGTTCC‐3′, Reverse: 5′‐AGTTTACGGAAGTCGGCGAG‐3′; GSDME: Forward: 5′‐ACATGCAGGTCGAGGAGAAGT‐3′, Reverse: 5′‐TCAATGACACCGTAGGCAATG‐3′; NLRP3: Forward: 5′‐GCTGGCATCTGGATGAGGAA‐3′, Reverse: 5′‐TGCCATCTTGACCCATCAGC‐3′; Caspase1: Forward: 5′‐ATCCGTTCCATGGGTGAAGG‐3′, Reverse: 5′‐GATGTGGGCATAGCTGGGTT‐3′; GAPDH: Forward: 5′‐GAAGGTGAAGGTCGGAGTC‐3′, Reverse: 5′‐GAAGATGGTGATGGGATTTC‐3′; 18S: Forward: 5′‐GTAACCCGTTGAACCCCATT‐3′, Reverse: 5′‐CCATCCAATCGGTAGTAGCG‐3′.

### CCK8 Analysis

Cell proliferation assays were determined by Cell Counting Kit‐8(CCK8, Proteintech, Wuhan, China). Cells were transfected with designed plasmids and/or siRNA and cultured into 96‐well plates at 3000 cells per well at 37 °C for 0, 24, 48, and 72 h. Afterward, 100 µL serum‐free culture medium containing 10 µL CCK‐8 reagent were added into each well and incubated for 2 h. The optical density (OD) value of CCK‐8 was evaluated at a wavelength of 450 nm by a Multiskan Spectrum 1500 (Thermo Labsystems, MA, USA).

### Colony Formation Assay

Treated MKN45 and HGC27 cells were seeded into 6‐well plates at a density of 600 cells per well. They were grown in fresh complete medium for ≈2 weeks and renewed every three days. Next, cells were fixed with 4% paraformaldehyde, stained with 0.2% crystal violet (Sigma‐Aldrich), and counted the number of colonies.

### Immunofluorescence

MKN45 and HGC27 cells were seeded on coverslips in 12‐well plates after being transfected with corresponding plasmids and/or siRNAs. When the cells adhered to the bottom of the slips and were grown at a density of 80%, the medium was discarded and washed with PBS twice times. In the next, fixed with 4% paraformaldehyde for 30 minutes, permeabilized with 0.5% Triton X‐100 and blocked with Goat serum (ZSGB‐BIO, Beijing, China) for one hour, cells were immunostained with GSDMD‐NT (1:200 dilution, ABclonal), HIC1(1:200, Santa Cruz), Jab1(1:200, Santa Cruz), CD8a (1:200, Proteintech) at 4 °C overnight. Followed by cells were incubated with Alexa Fluor 555‐conjugated goat anti‐rabbit ‐IgG (Invitrogen) for one hour at room temperature. Finally, 4–6‐diamidino‐2‐phenylindole (DAPI, Beyotime) was used to stain nuclei and a Cell Plasma Membrane Staining Kit with Dil (Red Fluorescence) (Dil, C1991S, Beyotime) was applied for observing the morphology of membrane in detail. Immunofluorescent photographs were captured by a fluorescence microscope (ZEISS, China).

### Transmission Electron Microscope (TEM) Assay

The slices of MKN45 cells were immobilized in 4% glutaraldehyde at 4 °C overnight, rinsed with 0.1 M PBS for 15 mins three times, and fixed with 1% osmic acid for 2 h. Subsequently, washing with PBS again, the slices were dehydrated using gradient ethanol and embedded. At last, slices stained with 1% uranyl acetate and lead citrate were made and photographed under transmission electron microscopy (JEM‐1230).

### Enzyme‐Linked Immunosorbent Assay (ELISA)

Human IL‐18 ELISA Kit (Elabscience Biotechnology Co., Ltd) and Human IL‐1β ELISA Kit (Elabscience Biotechnology Co., Ltd) were applied to measure IL‐18 and IL‐1β concentrations in the cell supernatants from MKN45 and HGC27 cells according to the manufacturer's instructions. In addition, transplanted tumor models of MKN45‐challenged C57BL/6J were established and tumors were harvested after euthanasia. Subsequently, grinding, centrifuging, and collecting, the supernatants were detected by the Mouse Granzyme B, Perforin1, Tumor Necrosis Factor Alpha (TNF‐α), Interferon‐gamma (IFN‐γ) through ELISA Kit (Elabscience Biotechnology Co., Ltd). Ultimately, the absorbance was determined at 450 nm by a spectrophotometer.

### TUNEL Assay

GC cells, with corresponding treatments, were plated on the coverslips into 12‐well plates for 24 h. Cells were fixed with 4% paraformaldehyde and exposed to 0.2% Triton‐X‐100 for permeabilization. Terminal deoxynucleotidyl transferase‐mediated nick end labeling (TUNEL) was measured by the Dead End Fluorometric TUNEL system (Promega, G3250). Washing with PBS, TUNEL, and DAPI fluorescent dyes were used for staining. The pyroptotic cells exhibiting green nuclear fluorescence were visualized using a fluorescence microscope.

### Cytotoxicity Assay

Cytotoxicity was measured by a Lactate dehydrogenase (LDH) release kit (Beyotime Biotech, C0017). The experiment was conducted in accordance with the manufacturer's instructions. Cells were seeded into 96‐well plates and set out to treat when the cell confluency arrived at 90%. After centrifugation at a speed of 400 rcf for 5 min, 120 µL supernatant of the individual well was transferred to another new 96‐well plate. Next, 60 µL of prepared LDH test working solution was added and incubated on the shaker in the dark for 30 min before measuring at 490 nm by a microplate reader.

### Propidium Iodide (PI) Staining

Propidium iodide (PI) staining assay was generally used to evaluate the integrity of the cell membrane. Briefly, cells were digested, centrifugated, and stained with 5 µL PI by PI detection kit (C1067 M, Beyotime, Shanghai, China). Thereafter, dripping onto the slips, the positive cells were photographed immediately and analyzed by Image J.

### Trypan Blue Staining

Briefly, Trypan blue staining was considered as a symbol of the disruption of the cell membrane. In other words, it was also applied to observing the conditions of cells that were alive or dead. After staining with Trypan Blue Staining Cell Viability Assay Kit (Beyotime, C0011), relatively accurate quantification of cell viability could be achieved by counting directly or after taking pictures under the microscope.

### Co‐immunoprecipitation (Co‐IP) and Mass Spectrometry Analysis

For Co‐immunoprecipitation assay, transfected cells were lysed using IP/Western lysing solution (Beyotime Biotechnology, P0013), followed by co‐incubation with HIC1/Jab1 antibody or IgG on a rocker at 4 °C overnight prior to being immunoprecipitated with protein A/G agarose beads (Beyotime) for 2 h. Then, rinsing the beads three times at a speed of 14 000 rpm for 1 min, cellular supernatant was collected and used for electrophoresis, Coomassie Blue Staining (Servicebio) or silver staining (Biosharp). Afterward, the differential gel bands of interest were removed and cut into 1 cm approximately. Mass spectrometry (MS) analysis was used for identification to discover putative sites.

### In Vitro Ubiquitination Assay

For in vitro ubiquitination assay, recombinant human Jab1 (Proteintech, Ag25956), ubiquitin (ABclonal, RPO1960), UBE1(R&D Systems, E‐305‐025), UBE2B (YEASEN, 20428es10) and ATP (Solarbio, C0550) were utilized. The lysate from cells overexpressing HIC1 was immunoprecipitated using an anti‐HIC1 antibody. The captured beads were then incubated with E1, E2, recombinant Jab1, ubiquitin, ATP, and ubiquitination buffer at 37 °C for 6 h. Following three washes with PBS, the ubiquitination status was accessed by western blot analysis.

### Chromatin Immunoprecipitation (ChIP) Assay

The EZ‐ChIP kit (Upstate, NY, USA) was performed in MKN45 and HGC27 cells according to the manufacturer's instructions. Treated cells were cross–linked with formaldehyde on the shaker at room temperature for 10 min and 125 mm glycine was added to quench this reaction. Afterward, cell extracts were shattered at a fragment of 200–300 bp with ultrasound and immunoprecipitated with HIC1 antibody or rabbit IgG as the negative control. Enrichment of the DNA template was measured by RT‐qPCR using primers specific for GSDMD promoter. The primers were as listed: P1: Forward: 5′‐CAGGGAGCAACACTCCTTCC‐3′, Reverse: 5′‐GGCTTACACCCGCTCCTG‐3′; P2: Forward: 5′‐CCAGGAGCGGGTGTAAGC‐3′, Reverse: 5′‐CAGGGCTTTGGGCGTCTG‐3′; P3: Forward: 5′‐GAGGCTGCCAGGACATAGTG‐3′, Reverse: 5′‐AGAGAGGTAATCCCCGAGCC‐3′; P4: Forward: 5′‐CTGGCAGGCACCTGAAGTC‐3′, Reverse: 5′‐CCAAGACAAGCGCTAGGGTG‐3′.

### Dual‐Luciferase Reporter Gene Assay

The dual‐luciferase reporter gene kit assay was carried out to investigate the activity of GSDMD promoter. The human GSDMD promoter and specified mutant plasmids were provided by IBOSI (Shanghai). And wild type, mutant, and deleted plasmids were constructed and inserted into PGL3‐basic luciferase‐reporter plasmid. According to the manufacturer's instructions, MKN45 and HGC27 cells were co‐transfected with control or HIC1 plasmids, along with pGL3 promoter constructs and a Renilla plasmid. After 6 h of transfection, the cells were incubated for an additional 36 h in a fresh complete medium. Following this, the cells were washed with PBS and lysed using luciferase assay buffer. Luciferase activity was subsequently measured using the Renilla‐Firefly Luciferase Dual Assay Kit (MCE, Cat. No.: HY‐K1013). The results were presented as the ratio of firefly luciferase activity to Renilla luciferase activity.

### Animal Studies

The ethical regulations were followed and all animal experimental procedures were conducted in agreement with the Animal Committee of Xuzhou Medical University (202211S026). To establish the subcutaneous GC model, male 5–6 weeks old BALB/C nude mice and C57BL/6J mice were provided by Nanjing Jicui Yaokang Bioscience (Nanjing, China). They grew up in a pathogen‐free environment with free access to food and water. After one week, the mice were randomly assigned into three groups (*n* = 6, per group). Transfected cells (5 × 10^6^ cells resuspended in 100 µL PBS mixed with Matrigel (1:1) (BD Biosciences)) were injected into the right hips. Dimethylfumarate (DMF) was administered intraperitoneally (i.p.) (10 mg kg^−1^, once every three days) when the tumor size approached 50–100 mm^3^ to assess the role of pyroptotic inhibitor in tumor growth. All mice were tracked individually using digital calipers for tumor size and the weight was recorded once every three days. In addition, the tumor volume was determined with the following formula: 0.5×length×width^2^. Meanwhile, to further access the function of the immune system, 615 line mice were purchased from Beijing HFK Bioscience Co., LTD (Beijing, China). 615 line mice (5 weeks old, 20–22 g weight, male) were used for xenograft models. MFC cells (5 × 10^5^) were subcutaneously injected into the right hips of the mice. Meanwhile, mouse‐PD‐L1 mAb (Bio Cell, B7‐H1) was utilized in LV‐HIC1 tumor‐bearing mice to evaluate whether HIC1 overexpression enhanced the effect of PD‐L1 mAb therapy. In vivo mAb treatments were performed by intraperitoneal injection (100 µg per mouse), combination therapy, or vehicle only, every three days for up to two weeks. At the end of the animal experiments, the BALB/C nude mice were euthanized and the tumor xenografted were harvested for immunohistochemical staining. And the single‐cell suspension of C57BL/6J mice tumor cells was prepared for FACS analysis.

### Immunohistochemistry

Mouse tumor specimens and human gastric cancer tumors and paired normal samples were made as 5 µm formalin‐fixed, paraffin‐embedded (FFPE) sections. The slides were deparaffinized, rehydrated, and incubated with primary antibodies (GSDMD/ HIC1/ Ki‐67) (1:200, ABclonal; 1:200, Proteintech; 1:100, Santa Cruz, respectively) at 4 °C overnight, followed by staining with a biotin‐conjugated secondary antibody. Finally, diaminobenzidine (DAB; Zhongshan Biotech, Beijing, China) was used for visualizing the target protein before hematoxylin counterstaining and scanned under a light microscope. To evaluate the immunohistochemistry results, two pathologists scored impartially according to the staining intensity and the percentage of positive cells. Specifically, staining intensity ranged from 0 to 3: 0: negative; 1: weak; 2: moderate; 3: strong. The percentage of positive cells was similarly scored into 4 categories: 1: (0–25%); 2: (26%–50%); 3: (51%–75%) and 4 (76%–100%) and were used to generate an immunoreactive score (IRS), which was calculated by multiplying the scores of staining intensities and the percentage of positive cells. Generally speaking, a score < 6 was defined as a low expression, and a score ≥ 6 was considered as high expression.

### Flow cytometry Analysis

At the end of the experiments, primary tumors removed from treated C57BL/6J mice were collected and the single‐cell suspension was derived by stripping gently, grinding physically, and filter filtration. Subsequently, centrifugated and blocked, cells were stained with antiCD45 (0.5 µg each test, Invitrogen #2452789), antiCD8a (0.125 each test, Invitrogen #2452838), antiIFN‐γ (0.5 each test, Invitrogen #2527204), antiPD‐1 (1 each test, Invitrogen #2366485), and antiCD3e (1 each test, Invitrogen #2516049) for 30 min at 4 °C avoid lighting. Washing with buffer three times, cells were resuspended by 500 µL buffer again and detected by a flow cytometer (BD FACSVerse).

### Immune Infiltration Analysis

TIMER web server (http://timer.comp‐genomics.org/) represents a comprehensive resource for systematical analysis of immune infiltrates in a wide range of cancer types. Here, the TIMER database was specifically dedicated to accessing the correlation between HIC1 and immune infiltrates in gastric cancer, encompassing B cells, CD4^+^ T cells, CD8^+^ T cells, Neutrophils, Macrophages, and Dendritic cells from the TIMER algorithm. Additionally, TISIDB (http://cis.hku.hk/TISIDB/), an integrated platform that consolidates diverse data types, serves as a valuable resource for exploring the intricate interplay between tumors and the immune system. Leveraging these databases, an in‐depth analysis was conducted to investigate the relationship between the abundance of immunomodulators and the expression levels of HIC1.

### Statistical Analysis

Data were presented as the mean ± SD. Student's t‐test and one‐way analysis of variance (ANOVA) test were applied for statistical comparisons between two independent groups or among multiple groups, respectively. Overall survival analysis was accessed by the Kaplan–Meier method, and survival curves divided into “high expression” and “low expression” were calculated using the log‐rank test. The Chi‐square test was utilized to analyze the percentage of HIC1, and GSDMD expression in GC and normal tissues. All statistical analysis was performed using GraphPad Prism software version 9 (San Diego, CA, USA) and Statistical Package for Social Sciences Version 21.0 (SPSS 21.0). A *p*‐value <0.05 was considered to be significant.

## Conflict of Interest

The authors declare no conflict of interest.

## Author Contributions

M.K., W.D., and L.D. contributed equally to this work. M.K. and W.D. performed the experiments. W.D. and M.W. analyzed the data. M.K., W.D., and L.D. wrote the paper. D.P. designed the study; All authors read and approved the final paper.

## Supporting information



Supporting Information

Supporting Information

## Data Availability

The data that support the findings of this study are available from the corresponding author upon reasonable request.
