# Supplementary material for: HIC1 suppresses Tumor Progression and Enhances CD8+ T Cells Infiltration Through Promoting GSDMD‐induced Pyroptosis in Gastric Cancer
Source: Adv Sci (Weinh). 2025 Apr 25;12(26):2412083. doi: 10.1002/advs.202412083 (PMC12245036; doi:10.1002/advs.202412083)
Supplement: Supplementary file 1 — Supporting Information [file ADVS-12-2412083-s001.docx]

**Supplementary figures**
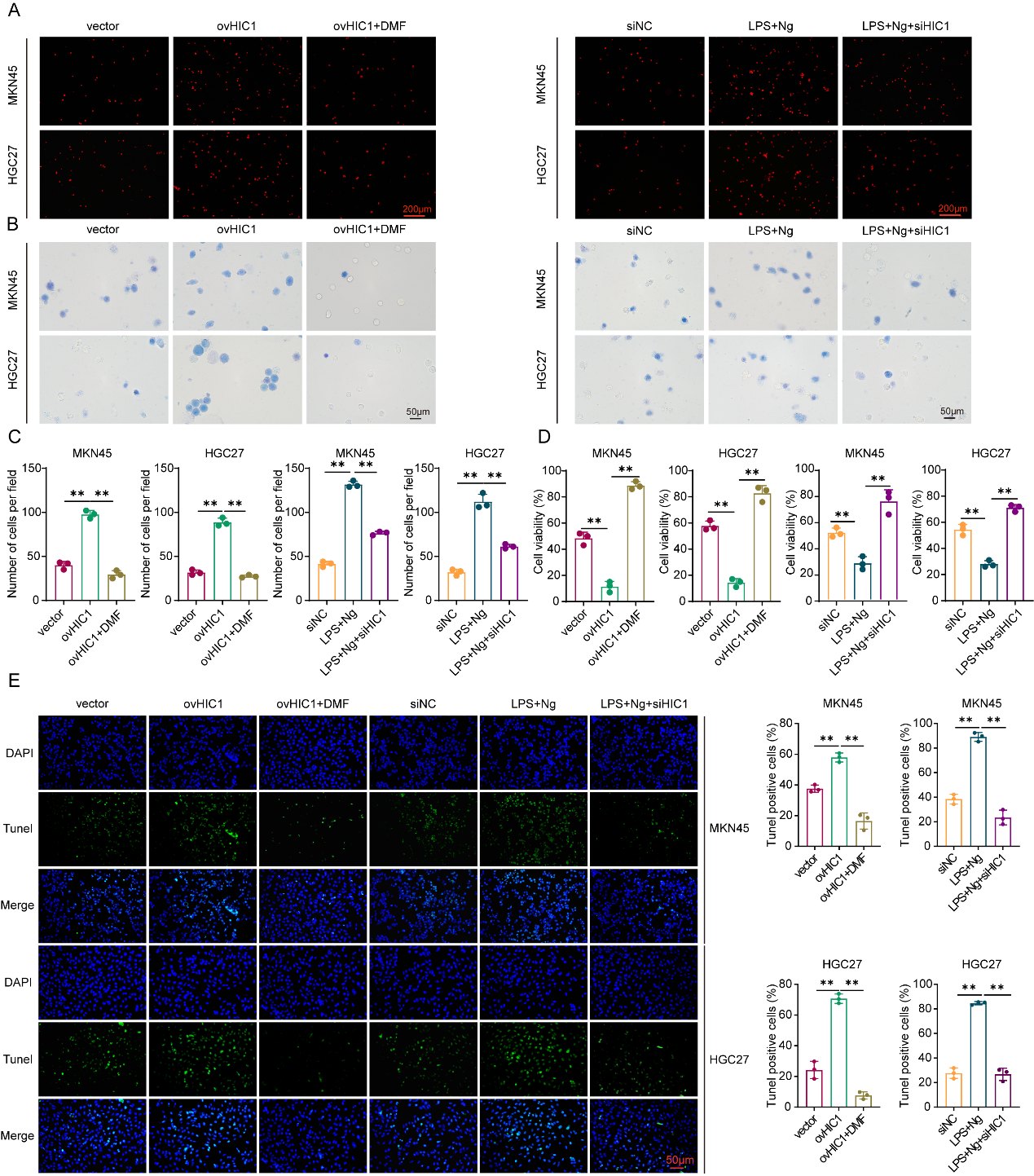


**Figure S1. HIC1 induces GSDMD-dependent pyroptosis.** (A) Representative images of PI staining of the vector group, ovHIC1 group, ovHIC1+DMF group, LPS+Ng group and LPS+Ng+siHIC1 group. Red fluorescence represents positive cells. Scale bars: 200μm. (B) Trypan blue staining was applied to measure the cell membrane integrity and vitality. And the treatment group was in accordance with the aforementioned. (C) TUNEL experiment and corresponding analysis were presented. Pyroptotic cells were showed in green and the nucleus was represented in blue. All experiments were indicated as mean ± SD. **P < 0.01.


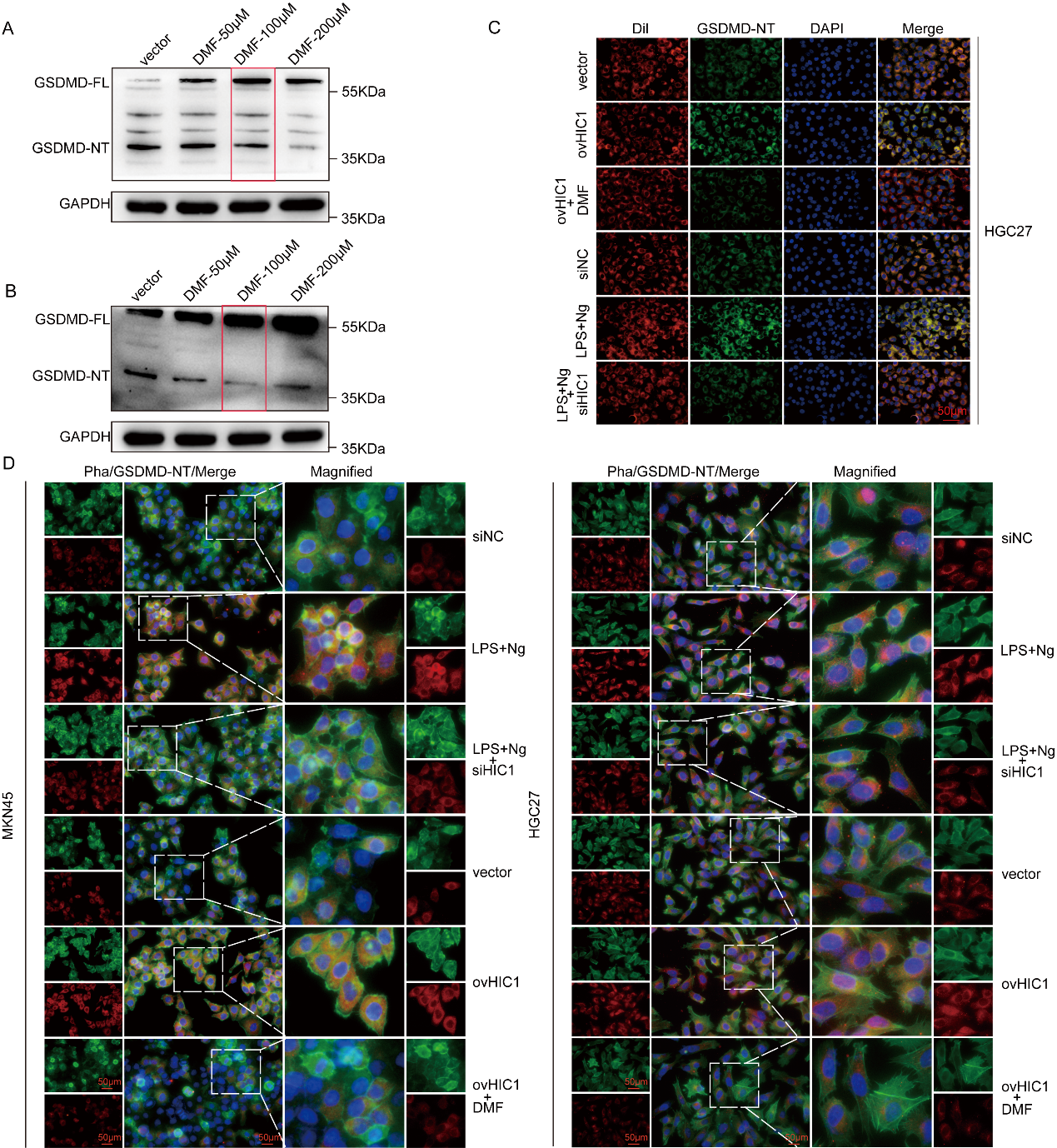


**Figure S2. HIC1 elevates the protein level of GSDMD-NT.** (A-B) MKN45 and HGC27 cells were administrated with various concentrations of DMF for 24h, GSDMD-FL and GSDMD-NT protein expression were determined by Western blot analysis. (C) Representative images of expression of GSDMD-NT in the membrane (magnification 400×) after cells were permeated. (D) Using fluorescence microscope, the alteration of fluorescence intensity of GSDMD-NT in the cytoplasm was showed and phalloidin was stained to observed the morphology in GC cells without permeating.


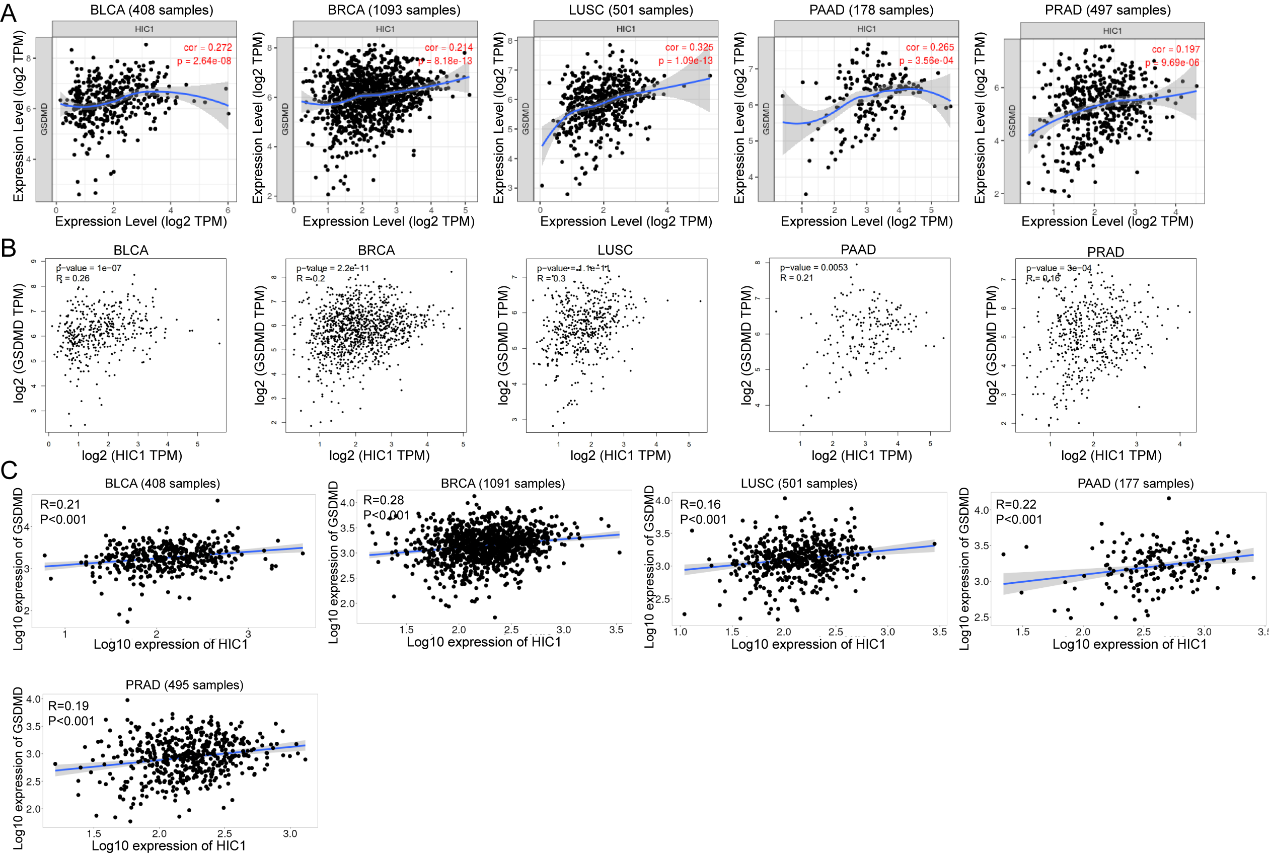


**Figure S3. HIC1 is positively associated with GSDMD across multiple databases.** (A) Correlation analysis of HIC1 and GSDMD was conducted through TIMER database. (B-C) The relationship between HIC1 and GSDMD in various malignancies, including BLCA, BRCA, LUSC, PAAD, and PRAD, was explored using the GEPIA (<http://gepia.cancer-pku.cn>) and TNMplot database.


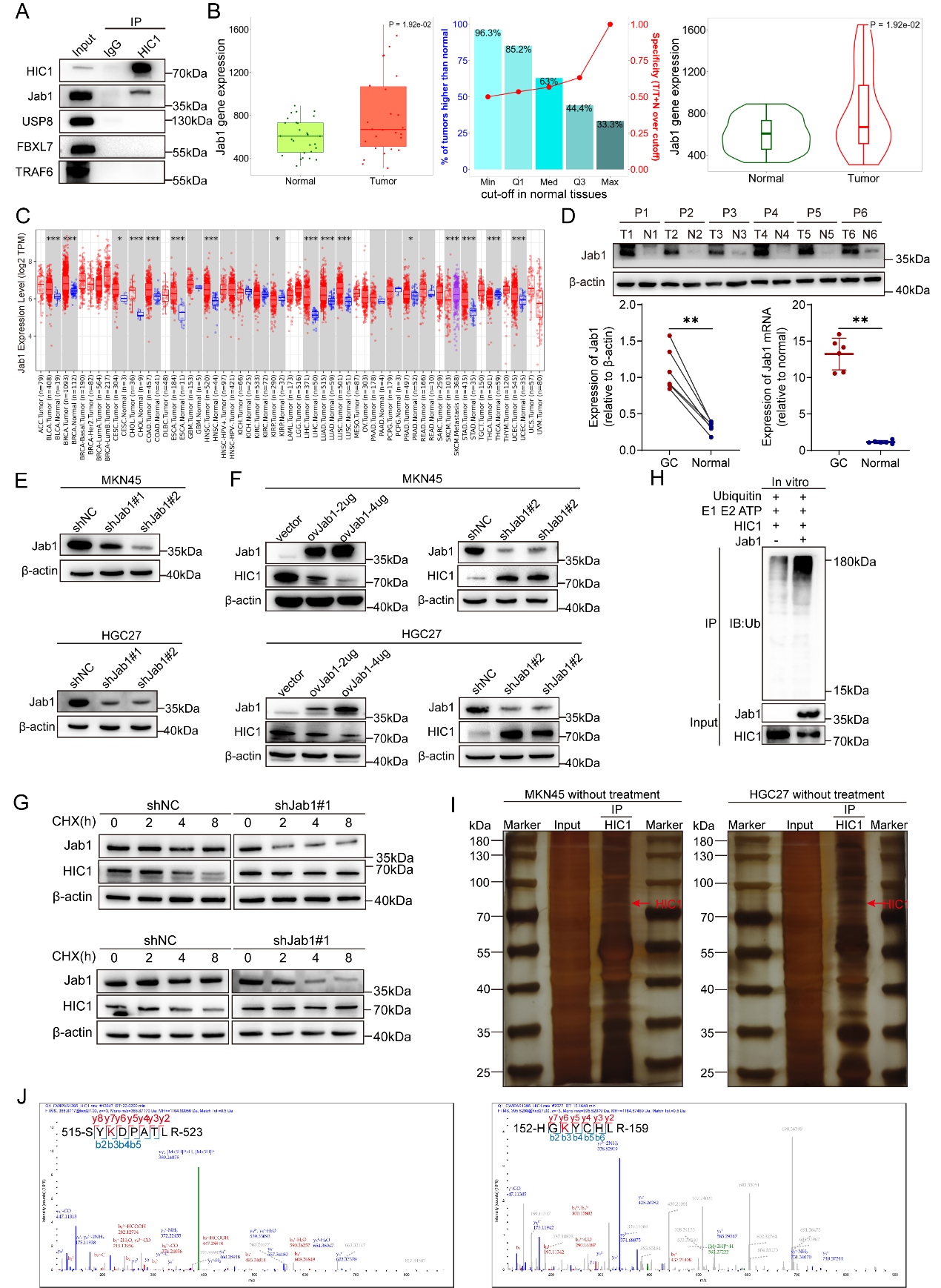


**Figure S4. Identification of Jab1 as a potential upstream of HIC1.** (A) Co-IP was performed to screen the upstream target of HIC1. (B-C) TNMplot and TIMER databases showed that Jab1 expression was higher in GC than normal tissues. (D) Western blot and RT-qPCR analysis were in line with bioinformation databases. (E) Two independent shJab1 were adopted to minimize off-target effects. (F)Western blot was used to evaluate the levels of HIC1 protein after overexpression in a dose-dependent manner or knockdown of Jab1 in MKN45 and HGC27 cells. (G) The levels of HIC1 protein were accessed by western blot following treatment with CHX. (H) In vitro ubiquitination assay. GC cells treated with HIC1 were incubated with recombinant Jab1 protein in the presence of E1, E2, ubiquitin, and ATP. Western blot was applied to examine the levels of ubiquitin. (I) Silver-stained image of HIC1 in GC cells. (J) Schematic diagram of mass spectrometry analysis of HIC1 at K517 and K154.


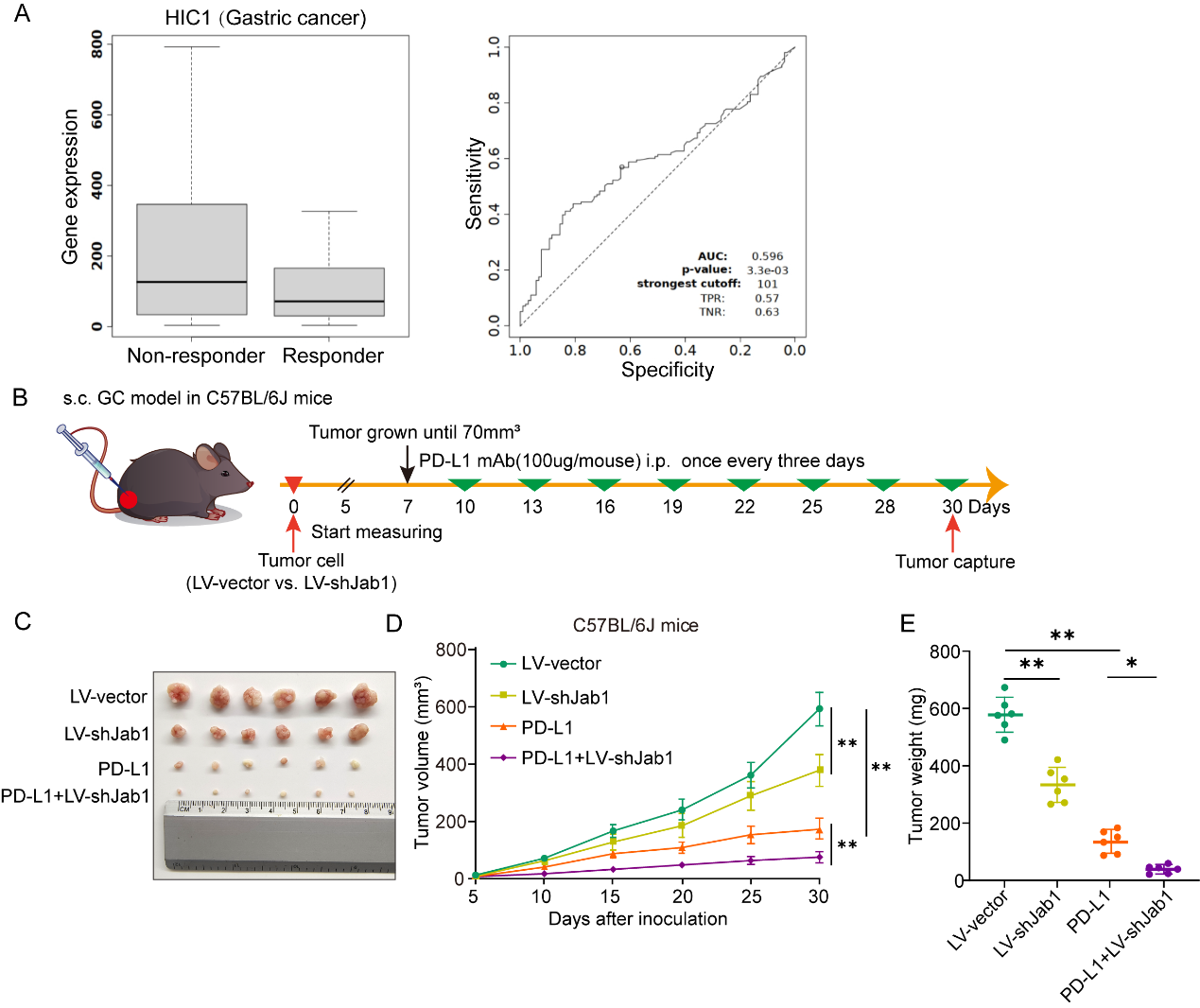


**Figure S5. HIC1 might act as a predictive biomarker for immunotherapy response.** (A) Using the ROC Plotter (https://rocplot.com/immune) database, the correlation between HIC1 expression levels and the therapeutic efficacy of PD-1 in gastric cancer was evaluated. (B) The schematic diagram of GC model in C57BL/6J mice. (C) Figures of tumors extracted from models (n=6). (D-E) Tumor growth curves and weight of the tumors for indicated groups.


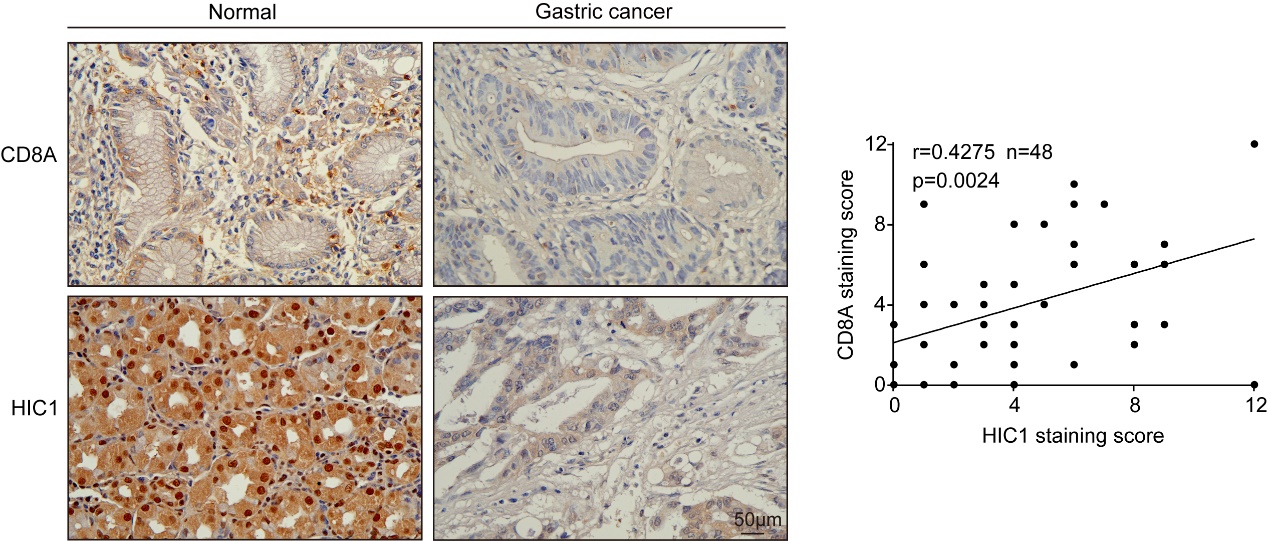


**Figure S6. Correlation between HIC1 and CD8A expression in clinical surgical specimens.** Immunohistochemical staining results were presented on the left (Normal group = 48, GC group = 48), with statistical analysis of the two proteins displayed on the right (r = 0.4275, n = 48, p=0.0024).
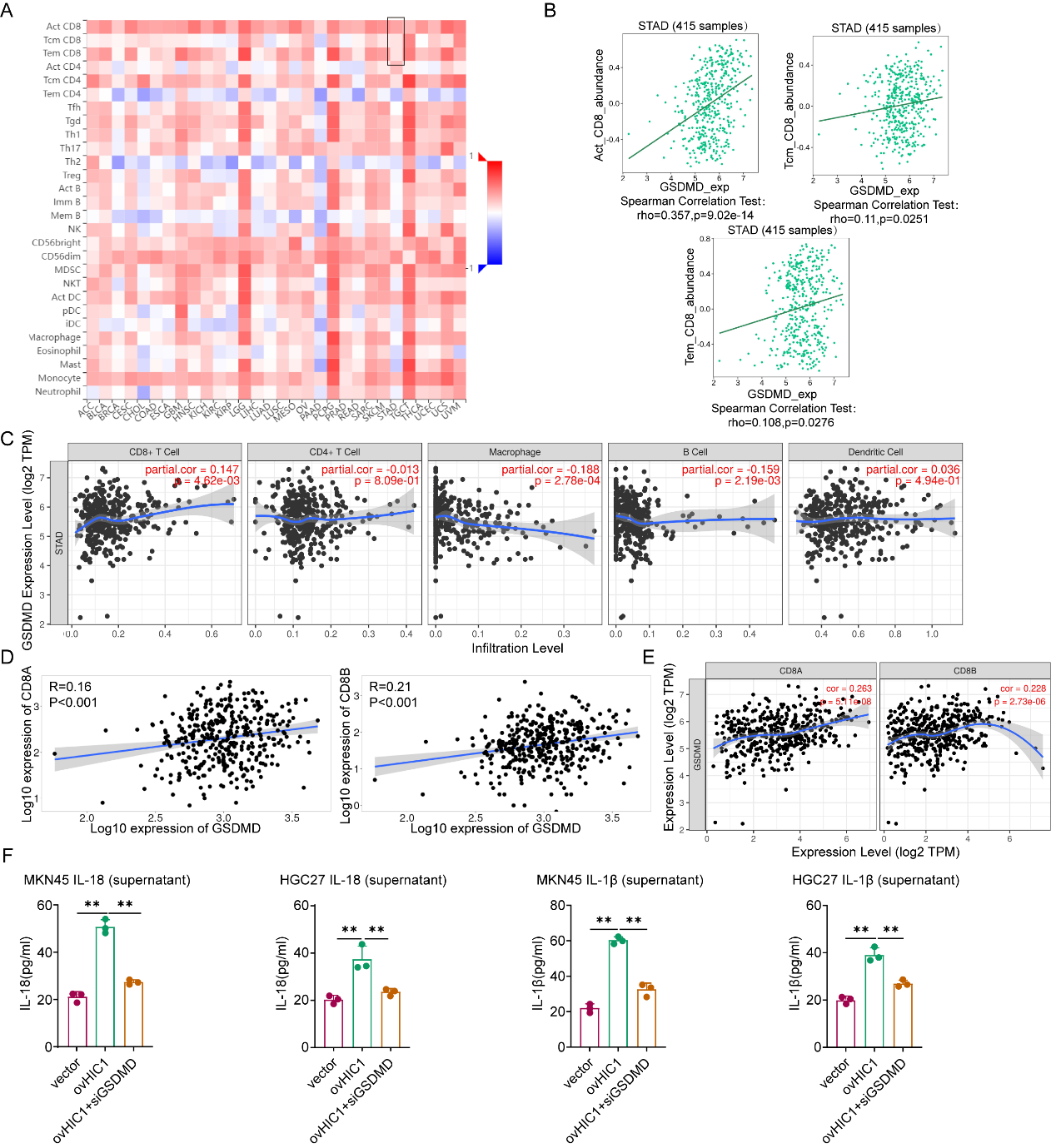


**Figure S7. GSDMD participates in the regulation of cytotoxic T cell function and pyroptosis.** (A-B) Bioinformatics analysis of the relationship between GSDMD and immune effector cells (Act_CD8, Tcm_CD8, and Tem_CD8) derived from TISIDB database. (C) Correlation between GSDMD expression and the infiltration of CD8^+^ T cell, CD4^+^ T cell, macrophage, B cell, and dendritic cell in gastric cancer. (D-E) Correlation between GSDMD expression and CD8A/B in gastric cancer. (F) ELISA assays were performed on MKN45 and HGC27 cells treated with HIC1 overexpression or GSDMD knockdown.


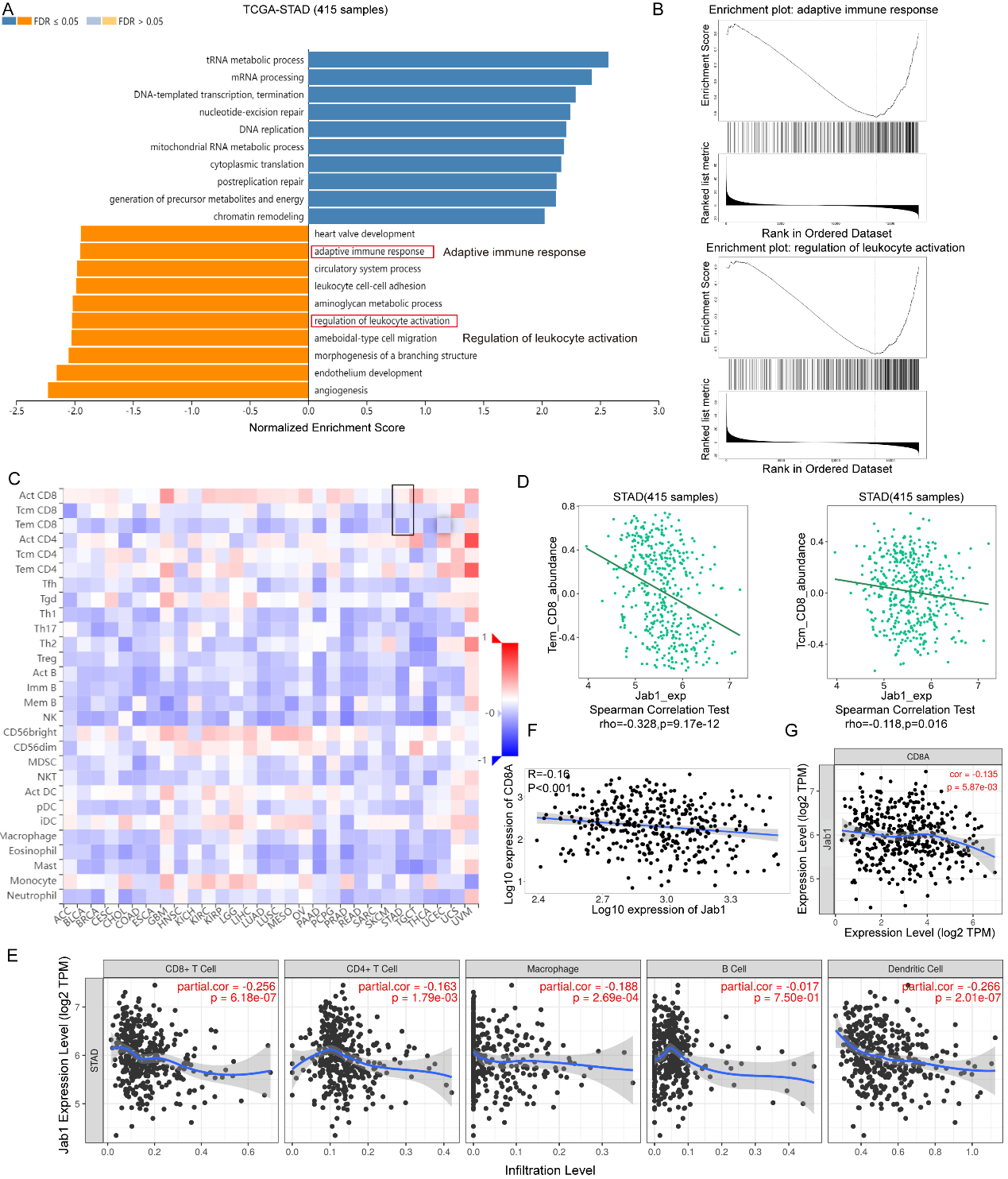


**Figure S8. Jab1 is implicated in cytotoxic T cell function.** (A-B) GO biological process analysis and GESA of Jab1-associated differential genes. (C-D) Relations between abundance of tumor-infiltrating lymphocytes (TILs) and Jab1 expression across various human cancers by TISIDB database. (E) Relations between Jab1 expression and the infiltration of CD8^+^ T cell, CD4^+^ T cell, macrophage, B cell, and dendritic cell in gastric cancer. (F-G) Correlations between Jab1 and CD8A expression in gastric cancer tissues.


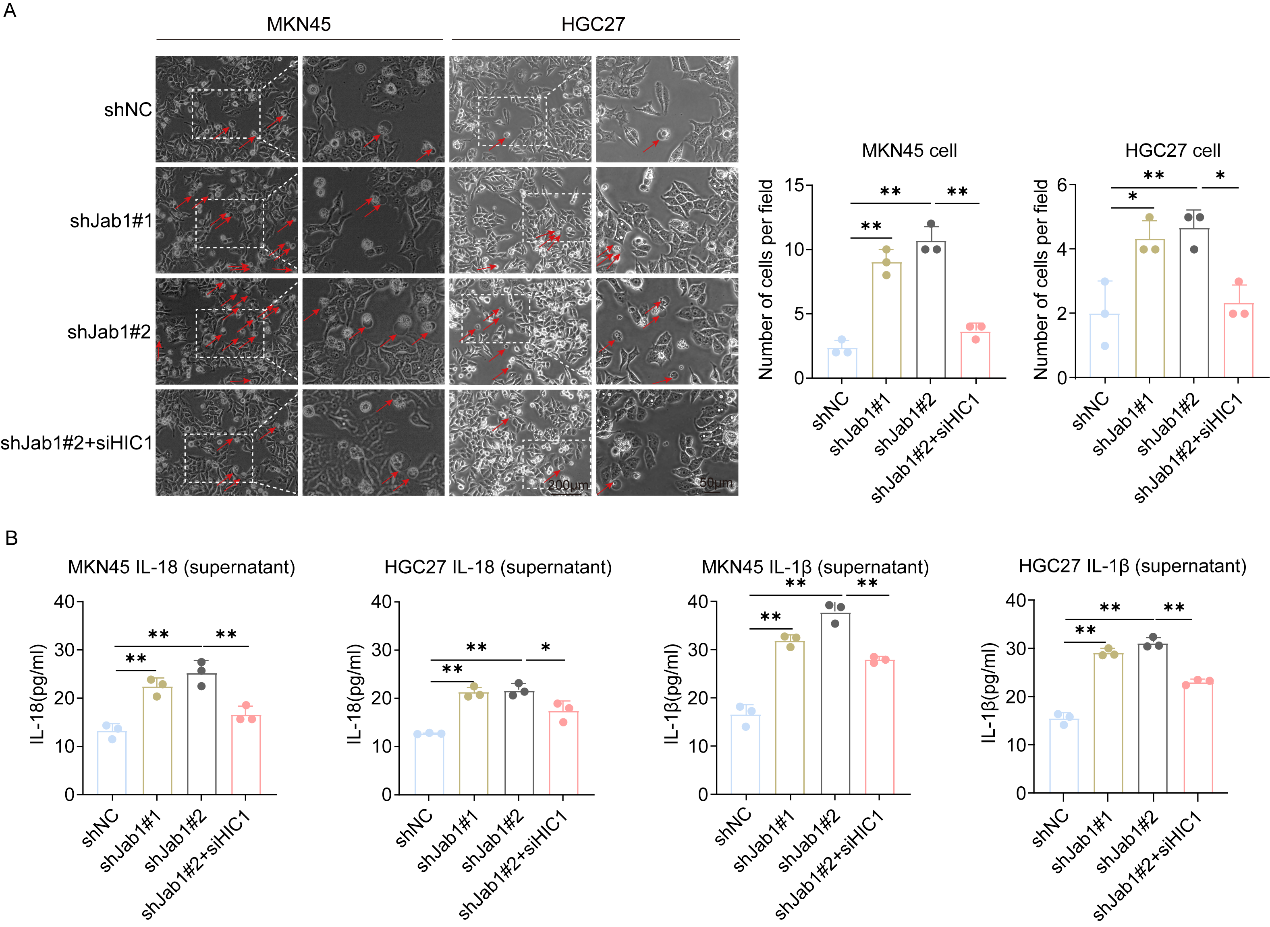


**Figure S9. Jab1 promotes the occurrence of pyroptosis.** (A) Representative light microscopy images of the shNC group, shJab1#1 group, shJab1#2 group and shJab1#2+siHIC1 group. (B) The secretion levels of IL-18 and IL-1β in the cell supernatant were quantified using ELISA assays. *P < 0.05, **P < 0.01.
