# Supplementary material for: HIC1 suppresses Tumor Progression and Enhances CD8+ T Cells Infiltration Through Promoting GSDMD‐induced Pyroptosis in Gastric Cancer
Source: Adv Sci (Weinh). 2025 Apr 25;12(26):2412083. doi: 10.1002/advs.202412083 (PMC12245036; doi:10.1002/advs.202412083)
Supplement: Supplementary file 2 — Supporting Information [file ADVS-12-2412083-s002.docx]

| **Variable** | **Low expression**  **of HIC1 (n=54)** | **High expression**  **of HIC1 (n=43)** | **Total**  **(n=97)** | ***P* value** |
| --- | --- | --- | --- | --- |
| **Gender** |  |  |  |  |
| Female | 14 | 11 | 25 | *P*=0.9497 |
| Male | 40 | 32 | 72 |  |
| **Age** |  |  |  |  |
| ≤60 years | 22 | 15 | 37 | *P*=0.3927 |
| >60 years | 32 | 28 | 60 |  |
| **Tumor size (cm)** |  |  |  |  |
| ≤5 | 42 | 35 | 77 | *P*=0.4745 |
| >5 | 12 | 8 | 20 |  |
| **TNM stage** |  |  |  |  |
| Ⅰ-Ⅲ | 32 | 35 | 67 | ***P*=0.0003** |
| Ⅳ | 22 | 8 | 30 |  |
| **pT status** |  |  |  |  |
| pT_1_-pT_3_ | 17 | 7 | 24 | ***P*=0.0037** |
| pT_4_ | 37 | 36 | 73 |  |
| **pN status** |  |  |  |  |
| pN_0_ | 20 | 15 | 35 | *P*=0.7757 |
| pN_1_-pN_3_ | 34 | 28 | 62 |  |
| **pM status** |  |  |  |  |
| pM_0_ | 33 | 35 | 68 | ***P*=0.0009** |
| pM_1_ | 21 | 8 | 29 |  |

**Supplementary Tables**

**Table S1**. Association of HIC1 expression with clinicopathological parameters.

*P*<0.05 was considered statistically significant. Two-sided chi-square test was used.

| **Prognostic variables** | **Univariate analysis**  **HR 95%CI *P*-value** | | | **Multivariate analysis**  **HR 95%CI *P*-value** | | |
| --- | --- | --- | --- | --- | --- | --- |
| **Sex**  (Female vs Male) | 0.771 | 0.382-1.558 | 0.469 | 1.026 | 0.470-2.242 | 0.948 |
| **Age (years)**  (≤60 vs >60) | 1.080 | 0.592-1.970 | 0.801 | 0.601 | 0.286-1.262 | 0.179 |
| **Tumor size (cm)**  (≤5 vs >5) | 0.889 | 0.446-1.772 | 0.738 | 0.809 | 0.377-1.738 | 0.588 |
| **TNM stage**  (Ⅰ-Ⅲ vs Ⅳ) | 0.146 | 0.079-0.272 | <0.001 | 0.019 | 0.002-0.201 | 0.001 |
| **pT status**  (pT1-pT3 vs pT4) | 1.377 | 0.727-2.609 | 0.326 | 2.412 | 1.161-5.009 | 0.018 |
| **pN status**  (pN0 vs pN1-pN3) | 0.451 | 0.235-0.864 | 0.016 | 0.431 | 0.207-0.897 | 0.024 |
| **pM status**  (pM0 vs pM1) | 0.159 | 0.086-0.294 | <0.001 | 7.102 | 0.753-66.954 | 0.087 |
| **HIC1 expression**  (Low vs High) | 3.779 | 1.746-8.181 | 0.001 | 2.497 | 1.100-5.667 | 0.029 |

**Table S2**. Univariate and multivariate Cox analysis of prognostic markers for OS in GC patients.

HR: hazard ratio; 95% CI: 95% confidence interval; P-value < 0.05 was considered statistically significant.
